# Modelling and Computation in the Valuation of Carbon Derivatives with Stochastic Convenience Yields

**DOI:** 10.1371/journal.pone.0125679

**Published:** 2015-05-26

**Authors:** Shuhua Chang, Xinyu Wang

**Affiliations:** 1 Research Center for Mathematics and Economics, Tianjin University of Finance and Economics, Tianjin 300222, China; 2 Institute of Policy and Management, Chinese Academy of Sciences, Beijing 100190, China; Shanxi University, CHINA

## Abstract

The anthropogenic greenhouse gas (GHG) emission has risen dramatically during the last few decades, which mainstream researchers believe to be the main cause of climate change, especially the global warming. The mechanism of market-based carbon emission trading is regarded as a policy instrument to deal with global climate change. Although several empirical researches about the carbon allowance and its derivatives price have been made, theoretical results seem to be sparse. In this paper, we theoretically develop a mathematical model to price the CO_2_ emission allowance derivatives with stochastic convenience yields by the principle of absence of arbitrage opportunities. In the case of American options, we formulate the pricing problem to a linear parabolic variational inequality (VI) in two spatial dimensions and develop a power penalty method to solve it. Then, a fitted finite volume method is designed to solve the nonlinear partial differential equation (PDE) resulting from the power penalty method and governing the futures, European and American option valuation. Moreover, some numerical results are performed to illustrate the efficiency and usefulness of this method. We find that the stochastic convenience yield does effect the valuation of carbon emission derivatives. In addition, some sensitivity analyses are also made to examine the effects of some parameters on the valuation results.

## Introduction

As we all have known, climate change is one of the main environmental problems. Over the last decades, numerous scientific studies have proved that greenhouse gases such as carbon dioxide undoubtedly contribute to the climate change a lot. The accumulation of greenhouse gases, particularly CO_2_, contributes to the high concentration of solar energy in the air, and the greenhouse effects can be reflected by the increase of the number of extreme weather events, such as tsunamis, floods and droughts. The greenhouse gas emission increases so fast that the temperature in the atmosphere is rising, which is now a big threat to all the species on the earth, and leads to the glacier melting and the sea level rising. Thus, one of the tasks for the environmental management is to mitigate the above changes, that is to find a way to reduce the CO_2_ emission effectively.

In response to climate changes, the Kyoto Protocol proposed a carbon emission trading scheme to establish a market and reduce the concentration of GHG in the atmosphere. Several national and regional emission markets such as Chicago Climate Exchange (CCX) have been established. The European Union Emission Trading Scheme (EU ETS), which is the world’s largest single market for CO_2_ emission allowances, was also formed under the Kyoto Protocol in 2005 and is referred to as a cap-and-trade system. The cap means the pre-allocated amounts of greenhouse gas that the emitter can release freely. It started with a period, 2005–2007, followed by the Kyoto commitment period 2008–2012. The third period will last from 2013 to 2020.

Emission permits trade of carbon is an environmental management method specifical for carbon dioxide emission, and it is an effective way of reducing emissions. In fact, it is estimated that the carbon emission permits trading implemented and to be planned will be able to reduce 330 million tons of carbon dioxide equivalent emissions each year at least, which accounts for about 7% of global annual emissions. America is the first to carry out the study on the theory of the emission trading. Croker [1] proposed a property method to control the air pollution, which has laid a theoretical foundation for emission permits trading. Montgomery [2] studies the joint cost minimization in a market equilibrium of competition, and proves theoretically that the market-based emission permits trading is superior to the traditional environmental governance policy obviously. Hahn [3] believes that the initial allocation of emission permits may influence the monopolistic behavior of sewage plants with the monopoly power, and influence further the market efficiency. In fact, the pollutant emission permits trading is always a means of local governments to implement environmental management. However, the carbon emission reduction problem is a global environmental problem, and the environmental effect is obviously cross-regional, so the carbon emission permits trading has become a global environmental problem. In this case, the old theories are no longer enough, and a series of new studies is required.

The basic idea of carbon emission permits trading, which is regarded as a key environmental management method, is to limit the total amount of emission by creating rights to emit a certain amount of carbon, which is called the cap, and to make these rights tradable. Because of the difference in the efficiency of using energy and emitting CO_2_ for different countries and regions, the emission permits have become a scarce resource under the quota system. Due to the scarcity of emission permits, the emitters have to bear higher costs for production, which should encourage them to reduce emissions as soon as possible. The management for emission permits trading is to analyze efficiently the costs of emission reduction, and utilize the markets to reach the goal of management. In the emission permits trading system, which has been established for the purpose of environmental management, the emitters can trade their own excessive permits.

In the studies of carbon trading based on the global environmental management in the past decades, Edwards and Hutton [4] simulate the allocation of carbon permits in the United Kingdom, and pointed out that the auctioning of emission permits can allow much of the potential ‘double dividend’ to be realized. Cramton [5] proposed that auction is a better way to allocate the cap of emission permits, as it distributes the costs flexibly, incents innovations, and reduces the contentious arguments among members. Klaassen [6] conducts three experiments for the six largest carbon emission regions by taking advantage of experimental economics, of which the first is a single bid auction and the second is a Walrasian, and the third relies on the bilateral trading. Moreover, the three measures can all capture a significant part of the potential cost savings of emission trading. Many disputes exist and some theoretical issues still need to be completed further by the scholars from all countries, of which the valuation of carbon derivatives is the most important.

The growing CO_2_ derivatives market attracts a wide range of industries and financial institutions. There are two meanings about such a market. On one hand, CO_2_ derivatives contracts meet the primary need of risk transfer from those who are risk averse to those who are willing to accept the risk of permits shortage situation. By hedging the price risk through carbon derivatives, buyers and sellers can make better plans about their businesses. On the other hand, the project investments, i.e. investments committed under the so-called Clean Development Mechanism (CDM) and Joint Implementation Mechanism (JIM), which return the CO_2_ emission reduction payoff that depends on the CO_2_ permit market price at a fixed period, can be considered as real option contracts. It is reasonable to regard these projects as contracts whose values depend on the CO_2_ permit spot prices. Similarly, the saved costs or revenue from the emission permits market may encourage the emitters to increase the technological abatement investments.

CO_2_ emission allowances markets have become more and more liquid, and may grow up to the most prosperous commodities markets around the world in the near future. Moreover, the literature is available for examining the CO_2_ allowance prices from the econometric or risk management angle. For instance, see the survey paper by Mnif and Davison [7]. Also, in [8] and [9] the dynamics of short-term price of CO_2_ emission allowances in EU ETS is studied by Benz and Truck, and some statistical properties is obtained. In [10], an empirical framework is proposed for the pricing and hedging of intra-phase and inter-phase futures and options on futures, in which it has been pointed out that for inter-phase futures, the cost-of-carry model is still applicable, but a stochastic and mean reverting convenience yield is needed for accurate pricing. In addition, the ADF, ECM-GARCH, and ECM-TGARCH models are used to probe the mean-reversion properties and volatility features of stochastic convenience yields for CO_2_ emissions allowances in [11]. Furthermore, see [12–14] for the empirical studies of risk premia in CO_2_ allowance spots and futures prices and the forecasting ability of volatility, respectively, and the evaluation of the progress of this market from the trial phase to the next commitment period (Phase II). Specially, it has been suggested that the marginal costs of abatement are equal for each company [15], and the equilibrium price for European Union Allowances (EUAs) equals the marginal costs of abatement [16].

However, to the best of our knowledge, there are very few theoretical studies in the previous literature for the valuation of carbon emission permits derivatives. From [11] and [10] we can see that the convenience yields for CO_2_ emission allowances show a mean-reverting process. Convenience yields are defined as the benefits or premiums associated with holding an underlying product or physical good, rather than the contract or derivatives product [17]. Spot holders can achieve potential benefits due to price volatility, but the holders of futures contracts can not attain such benefits. The pricing problem, which contains a stochastic convenience yield, can not be done as the one in a complete market. As we all know, the derivative can be replicated by a portfolio of existing assets such as stocks and bonds, and the risk exposure can be eliminated in a complete market, while it is not valid in an incomplete market. Schwartz et al. [18, 19] and Hilliard [20] have studied the valuation of oil contingent claims and other derivatives with stochastic convenience yield. Besides, from the viewpoint of game theory, many researches have been done on how people make decisions to adapt climate change. See, for example, [21–28].

In order to bridge the gap between theory and practice, in this paper we theoretically derive the pricing partial differential equations of CO_2_ emission allowance derivatives, futures and options, with stochastic convenience yields by using the no-arbitrage principle and risk premium. Since the value of an American option is determined by a linear complementarity problem, a power penalty approach is proposed for the linear complementarity problem. That is, we will approximate the linear complementarity problem by a nonlinear parabolic PDE in two spatial dimensions with an *l*_*k*_ penalty term. In addition, a so-called finite volume method is presented for the numerical solution of the two-dimensional nonlinear partial differential equation. The innovation of this method is in that it combines a finite volume formulation with a fitted approximation. The finite volume method possesses a special feature of the local conservativity of the numerical flux, and is becoming more and more popular. See, for instance, Wang [29] for degenerate parabolic problems, Leveque [30] for hyperbolic problems, and Liu [31] for elliptic problems. Here we aim to present the theoretical valuation of carbon emission derivatives using partial differential equations coupled with numerical methods.

The paper is organized as follows. In Section 2, the pricing PDEs of the CO_2_ emission allowance derivatives are obtained, and the final and boundary conditions are prescribed for different contingent claims. Then, a power penalty method is introduced for the American option in Section 3. In Section 4, a so-called fitted volume method is proposed for the discretization of the pricing PDE. Some numerical experiments are performed to illustrate the efficiency and usefulness of the numerical method in Section 5. In addition, some sensitivity analyses are also made to examine the effects of some parameters on the valuation results in Section 6. Finally, some comments are given in Section 7.

## The pricing model of CO_2_ contingent claims

In order to achieve the pricing equation, which applies to any contingent claims for CO_2_ emission allowance spot prices, we assume that the spot price of CO_2_ emission allowance *S*_*t*_ at time *t* and the convenience yield *δ* follow a joint diffusion process specified by (see, for example, [10, 32])

$$\begin{array}{c} dS_{t}=(k_{s}(\mu \left(t\right)-\ln\left(S_{t}\right)+\frac{1}{k_{s}}\left(\frac{1}{2}\sigma_{s}^{2}+\frac{d\mu }{dt}\right))-\delta )S_{t}dt+\sigma_{s}S_{t}dW_{s}^{P}, \end{array}$$ (1)

$$\begin{array}{c} d\delta =k_{c}\left(\theta_{c}-\delta \right)dt+\sigma_{c}dW_{c}^{P}, \end{array}$$ (2)

where *k*_*s*_ and *k*_*c*_ denote the speeds of mean-reversion of spot price and convenience yield, *σ*_*s*_ and *σ*_*c*_ denote the constant volatilities of spot price and convenience yield, respectively, *θ*_*c*_ is the long-run mean yield, and *μ*(*t*) = ln (the marginal abatement cost) + *ξt* is the logarithm of the marginal abatement cost *C*_*s*_ growing at rate *ξ*. In addition, $dW_{s}^{P}$ and $dW_{c}^{P}$ are correlated increments to standard Brownian process under the historical measure ℙ, and they have the correlation coefficient *ρ*_*sc*_. From the concept of absence of arbitrage opportunities, if the market prices of spot and convenience yield are given, then we know

$$\begin{array}{c} W_{s}^{Q}=W_{s}^{P}+\int_{0}^{t}\lambda_{s}\left(\tau \right)d\tau \;\;\text{and}\;W_{c}^{Q}=W_{c}^{P}+\int_{0}^{t}\lambda_{c}\left(\tau \right)d\tau \end{array}$$

are Brownian motions under the risk-neutral measure ℚ, where *λ*_*s*_ and *λ*_*c*_ are the market prices of spot and convenience yield, respectively.

Note that Eq (1) is based on the relationship between the CO_2_ emission spot price and the marginal abatement cost given by

$$\begin{array}{c} \ln\left(S_{t}\right)=\mu \left(t\right)+X_{t}, \end{array}$$ (3)

where *X*_*t*_ is governed by an Ornstein-Uhlenbeck process with a zero long run mean, a speed of adjustment *k*_*s*_, and a constant volatility *σ*_*s*_:

$$\begin{array}{c} dX_{t}=-k_{s}X_{t}dt+\sigma_{s}dW_{s}^{p}. \end{array}$$

Eq (3) implies that the natural logarithm of the CO_2_ emission allowance spot price deviates around a mean-reversion level, and this level grows with the discount rate *ξ* and is determined by the marginal cost of abatement *C*_*s*_. Moreover, it is expected to be mean-reversed for convenience yields because of the strong tendency, and short-term random convenience yields converge to their mean values in the long run.

### The partial differential equation for the price of carbon derivatives

Assume that the price of carbon derivative *F*(*S*, *δ*, *t*) is a twice continuously differentiable function of *S* and *δ*, where we omit the subscript *t* of *S* for brevity. Then, we can use It$\hat{\text{o}}$’s lemma to derive the instantaneous price change as follows:

$$\begin{array}{ccc} dF & = & \frac{\partial F}{\partial t}dt+\frac{\partial F}{\partial S}dS+\frac{\partial F}{\partial \delta }d\delta +\frac{1}{2}\frac{\partial^{2}F}{\partial S^{2}}{\left(dS\right)}^{2}+\frac{1}{2}\frac{\partial^{2}F}{\partial \delta^{2}}{\left(d\delta \right)}^{2}+\frac{\partial^{2}F}{\partial S\partial \delta }dSd\delta \\ & = & (\frac{\partial F}{\partial t}+\left(k_{s}\left(n\left(t\right)-\ln S\right)-\delta \right)S\frac{\partial F}{\partial S}+k_{c}\left(\theta_{c}-\delta \right)\frac{\partial F}{\partial \delta }+\frac{1}{2}\sigma_{s}^{2}S^{2}\frac{\partial^{2}F}{\partial S^{2}}+\frac{1}{2}\sigma_{c}^{2}\frac{\partial^{2}F}{\partial \delta^{2}}\\ & & +\rho_{sc}\sigma_{s}\sigma_{c}S\frac{\partial^{2}F}{\partial S\partial \delta })dt+\sigma_{s}S\frac{\partial F}{\partial S}dW_{s}^{P}+\sigma_{c}\frac{\partial F}{\partial \delta }dW_{c}^{P}, \end{array}$$

from which the relative change can be obtained via dividing both sides by *F*

$$\begin{array}{ccc} \frac{dF}{F} & = & ((\frac{\partial F}{\partial t}+\left(k_{s}\left(n\left(t\right)-\ln S\right)-\delta \right)S\frac{\partial F}{\partial S}+k_{c}\left(\theta_{c}-\delta \right)\frac{\partial F}{\partial \delta }+\frac{1}{2}\sigma_{s}^{2}S^{2}\frac{\partial^{2}F}{\partial S^{2}}+\frac{1}{2}\sigma_{c}^{2}\frac{\partial^{2}F}{\partial \delta^{2}}\\ & & +\rho_{sc}\sigma_{s}\sigma_{c}S\frac{\partial^{2}F}{\partial S\partial \delta })/F)dt+(\sigma_{s}S\frac{\partial F}{\partial S}/F)dW_{s}^{P}+(\sigma_{c}\frac{\partial F}{\partial \delta }/F)dW_{c}^{P}, \end{array}$$

where

$$\begin{array}{c} n\left(t\right)=\mu \left(t\right)+\frac{1}{k_{s}}(\frac{1}{2}\sigma_{s}^{2}+\frac{d\mu }{dt}). \end{array}$$

For simplicity, we define

$$\begin{array}{ccc} k & = & (\frac{\partial F}{\partial t}+\left(k_{s}\left(n\left(t\right)-\ln S\right)-\delta \right)S\frac{\partial F}{\partial S}+k_{c}\left(\theta_{c}-\delta \right)\frac{\partial F}{\partial \delta }+\frac{1}{2}\sigma_{s}^{2}S^{2}\frac{\partial^{2}F}{\partial S^{2}}+\frac{1}{2}\sigma_{c}^{2}\frac{\partial^{2}F}{\partial \delta^{2}}\\ & & +\rho_{sc}\sigma_{s}\sigma_{c}S\frac{\partial^{2}F}{\partial S\partial \delta })/F, \end{array}$$ (4)

$$\begin{array}{ccc} L_{1} & = & (\sigma_{s}S\frac{\partial F}{\partial S})/F,\;L_{2}=(\sigma_{c}\frac{\partial F}{\partial \delta })/F. \end{array}$$ (5)

Following Brennan and Schwartz [33], we construct a portfolio *P* by investing amounts of *x*_1_, *x*_2_, and *x*_3_ in three options of maturities *τ*_1_, *τ*_2_, and *τ*_3_, respectively. Then, the rate of return on this portfolio is given by

$$\begin{array}{c} \frac{dP}{P}=\left[x_{1}k+x_{2}k+x_{3}k\right]dt+\left[x_{1}L_{1}+x_{2}L_{1}+x_{3}L_{1}\right]dW_{s}^{P}+\left[x_{1}L_{2}+x_{2}L_{2}+x_{3}L_{2}\right]dW_{c}^{P}, \end{array}$$ (6)

which is non-stochastic if the portfolio proportions are chosen so that the coefficients of $dW_{s}^{P}$ and $dW_{c}^{P}$ in Eq (6) are zero. That is,

$$\begin{array}{c} x_{1}L_{1}+x_{2}L_{1}+x_{3}L_{1}=0,\;x_{1}L_{2}+x_{2}L_{2}+x_{3}L_{2}=0. \end{array}$$ (7)

To eliminate the arbitrage opportunity, the above return on the portfolio must be risk-free over short time intervals, this is, the return rate is equal to *r*, the instantaneous risk-free interest rate. As a consequence, the portfolio risk premium is zero:

$$\begin{array}{c} x_{1}\left(k\left(\tau_{1}\right)-r\right)+x_{2}\left(k\left(\tau_{2}\right)-r\right)+x_{3}\left(k\left(\tau_{3}\right)-r\right)=0. \end{array}$$ (8)

The arbitrage free condition Eq (8) and the two zero risk conditions Eq (7) have a solution only if

$$\begin{array}{c} k-r=\lambda_{s}L_{1}+\lambda_{c}L_{2}, \end{array}$$ (9)

where *λ*_*s*_ and *λ*_*c*_ are the market prices of per unit spot and convenience yield, respectively.

Subsequently, substituting Eqs (4) and (5) into Eq (9) results in

$$\begin{array}{ccc} & & (\frac{\partial F}{\partial t}+\left(k_{s}\left(n\left(t\right)-\ln S\right)-\delta \right)S\frac{\partial F}{\partial S}+k_{c}\left(\theta_{c}-\delta \right)\frac{\partial F}{\partial \delta }+\frac{1}{2}\sigma_{s}^{2}S^{2}\frac{\partial^{2}F}{\partial S^{2}}+\frac{1}{2}\sigma_{c}^{2}\frac{\partial^{2}F}{\partial \delta^{2}}\\ & & \;+\rho_{sc}\sigma_{s}\sigma_{c}S\frac{\partial^{2}F}{\partial S\partial \delta })/F-r=\lambda_{s}(\sigma_{s}S\frac{\partial F}{\partial S})/F+\lambda_{c}(\sigma_{c}\frac{\partial F}{\partial \delta })/F. \end{array}$$

That is,

$$\begin{array}{ccc} & & \frac{\partial F}{\partial t}+\left(k_{s}\left(n\left(t\right)-\ln S\right)-\delta -\lambda_{s}\sigma_{s}\right)S\frac{\partial F}{\partial S}+\left(k_{c}\left(\theta_{c}-\delta \right)-\lambda_{c}\sigma_{c}\right)\frac{\partial F}{\partial \delta }+\frac{1}{2}\sigma_{s}^{2}S^{2}\frac{\partial^{2}F}{\partial S^{2}}\\ & & \;+\;\frac{1}{2}\sigma_{c}^{2}\frac{\partial^{2}F}{\partial \delta^{2}}+\rho_{sc}\sigma_{s}\sigma_{c}S\frac{\partial^{2}F}{\partial S\partial \delta }-rF=0, \end{array}$$ (10)

which is the partial differential equation for the contingent claims depending on the evolution of the spot price *S* and the convenience yield *δ*.

**Remark 1.** *In the case of futures contract, the return rate on the portfolio* Eq (6) *should be zero [* 34 *], such that the* Eq (8) *changes into* *x*_1_ *k*(*τ*_1_)+*x*_2_ *k*(*τ*_2_)+*x*_3_ *k*(*τ*_3_) = 0. *Then, the pricing partial differential equation becomes*

$$\begin{array}{ccc} & & \frac{\partial F}{\partial t}+\left(k_{s}\left(n\left(t\right)-\ln S\right)-\delta -\lambda_{s}\sigma_{s}\right)S\frac{\partial F}{\partial S}+\left(k_{c}\left(\theta_{c}-\delta \right)-\lambda_{c}\sigma_{c}\right)\frac{\partial F}{\partial \delta }+\frac{1}{2}\sigma_{s}^{2}S^{2}\frac{\partial^{2}F}{\partial S^{2}}\\ & & \;+\;\frac{1}{2}\sigma_{c}^{2}\frac{\partial^{2}F}{\partial \delta^{2}}+\rho_{sc}\sigma_{s}\sigma_{c}S\frac{\partial^{2}F}{\partial S\partial \delta }=0. \end{array}$$ (11)

**Remark 2.** *The same pricing partial differential equation can be also obtained by using two-dimensional version of the Feynman-Kac formula.*

### Boundary and final conditions for different carbon derivatives

It stands to reason that we should prescribe the final conditions for different derivatives to fully define the problem. In addition, to solve the pricing PDE numerically, we also need the boundary conditions. In this paper, we propose three kinds of derivatives, European and American options as well as futures, which are the popular derivatives in any financial market.

#### Options

We only take the call option’s final and boundary conditions into consideration, since the put option is similar. The most common final condition is the vanilla option’s payoff given by

$$\begin{array}{c} F\left(S,\delta ,T\right)=\max\;\left(0,S-K\right),\;S\in \left(0,S_{\max}\right), \end{array}$$ (12)

where *K* denotes the exercise price of the option satisfying 0 < *K* < *S*_max_. A second choice is the cash-or-nothing payoff given by

$$\begin{array}{c} F\left(S,\delta ,T\right)=Bℍ\left(S-K\right),\;S\in \left(0,S_{\max}\right), \end{array}$$ (13)

where *B* > 0 is a constant and ℍ denotes the Heaviside function. Obviously, this final condition is a step function, which is zero if *S* < *K* and *B* if *S* ≥ *K*.

The price of any derivatives except the futures contract will satisfy Eq (10), and the boundary conditions have to be adjusted according to the specific exercise features of derivatives. In the case of European options, there are four boundaries in the solution domain: *S* = 0, *S* = *S*_max_, *δ* = *δ*_min_, and *δ* = *δ*_max_. Obviously, the boundary conditions at *S* = 0 and *S* = *S*_max_ are simply taken to be the extension of the final condition:

$$\begin{array}{c} F\left(0,\delta ,t\right)=F\left(0,\delta ,T\right)=0\;\text{and}\;F\left(S_{\max},\delta ,t\right)=F\left(S_{\max},\delta ,T\right). \end{array}$$ (14)

In the case of American options, since they can be exercised at any point in time before maturity, part of the valuation problem involves identifying the optimal exercise policy, or the exercise time that maximizes the option value. The boundary condition at *S* = *S*_max_ should be replaced by the following two classic value-matching and smooth-pasting boundary conditions [35]:

$$\begin{array}{c} F\left(S^{⋆}\left(t\right),\delta ,t\right)=S^{⋆}\left(t\right)-K, \end{array}$$ (15)

$$\begin{array}{c} \frac{\partial F(S^{⋆}\left(t\right),\delta ,t)}{\partial S}=1, \end{array}$$ (16)

for the American call option.

To determine the boundary conditions at *δ* = *δ*_min_ and *δ* = *δ*_max_, we need to model the pricing Eq (10) again for two particular values *δ* = *δ*_min_ and *δ* = *δ*_max_, and solve the two resulting one-dimensional equations. These two approximate boundary conditions will be used to implement our numerical methods.

#### Futures

In the case of futures, the way to determine the boundary conditions is similar to that for European options. We ignore the discussion about boundary conditions and only propose the final condition in this subsection. As mentioned in [36], when the delivery period is being reached, the futures price is very close or even equal to the spot price, which means that the final condition for futures should be

$$\begin{array}{c} F(S,\delta ,T)=S. \end{array}$$ (17)

## The power penalty approach

Since the valuation of American option is a free boundary problem, its exact solution is not available analytically. Therefore, numerical approximation to the solution is normally sought in practice. In fact, we formulate the free boundary problem as a linear complementarity problem, and then develop a power penalty method to solve it. That is, a nonlinear parabolic PDE in two spatial dimensions with an *l*_*k*_ penalty term is utilized to approximate the linear complementarity problem. Moreover, in the next section, a fitted finite volume method is proposed to solve the penalized nonlinear equation.

### Formulation of the problem into a complementarity problem

Denote the value of the American option with expiry date *T* by *F*(*S*, *δ*, *t*) and define

$$\begin{array}{c} LF=-\frac{\partial F}{\partial t}-\frac{1}{2}(\sigma_{s}^{2}S^{2}\frac{\partial^{2}F}{\partial S^{2}}+2\rho_{sc}\sigma_{s}\sigma_{c}S\frac{\partial^{2}F}{\partial S\partial \delta }+\sigma_{c}^{2}\frac{\partial^{2}F}{\partial \delta^{2}})-(h\left(S,\delta ,t\right)\frac{\partial F}{\partial S}+g\left(\delta ,t\right)\frac{\partial F}{\partial \delta })+rF, \end{array}$$ (18)

where *h*(*S*, *δ*, *t*) = *S*(*k*_*s*_(*n*(*t*) − ln *S*) − *λ*_*s*_ *σ*_*s*_ − *δ*) and *g*(*δ*, *t*) = *k*_*c*_(*θ*_*c*_ − *δ*) − *λ*_*c*_ *σ*_*c*_. Here we define *h*(0, *δ*, *t*) = 0 to keep the continuity of *h*(*S*, *δ*, *t*) at *S* = 0. The free boundary *S* ^⋆^(*t*) divides the domain Ω = (0, *S*_max_) × (*δ*_min_, *δ*_max_) into the continuation region Σ_1_ and the stopping region Σ_2_. In the continuation region Σ_1_, *F* > *F*(*S*, *δ*, *T*), *LF* = 0; in the stopping region Σ_2_, note that *S* > *K*, *F* = *S* − *K*, then *LF* > 0. In a word, the American option value *F* satisfies the following partial differential complementarity problem:

$$\begin{array}{c} \left\{ \begin{array}{c} LF\geq 0,\\ F-F^{⋆}\geq 0,\\ LF\cdot (F-F^{⋆})=0, \end{array}\right. \end{array}$$ (19)

for (*S*, *δ*, *t*) ∈ Ω × [0, *T*) with the boundary conditions:

$$\begin{array}{c} \left\{ \begin{array}{c} F\left(0,\delta ,t\right)=0,F\left(S_{\max},\delta ,t\right)=S_{\max}-K,\\ \;F\left(S,\delta_{\min},t\right)=g_{1}\left(S,t\right),F\left(S,\delta_{\max},t\right)=g_{2}\left(S,t\right), \end{array}\right. \end{array}$$ (20)

and the terminal condition

$$\begin{array}{c} F\left(S,\delta ,T\right)=F^{⋆}\left(S,\delta \right), \end{array}$$ (21)

where

$$F^{⋆}(S,\delta )=\text{max(}S-K,0\text{)}$$

is the payoff function, *g*_1_, and *g*_2_ are the boundary conditions to be determined in the next section.

For the ease of theoretical analysis, we rewrite Eq (18) as the following divergent form:

$$\begin{array}{c} LF=-\frac{\partial F}{\partial t}-\nabla \cdot \left(A\nabla F+\underline{b}F\right)+\bar{c}F, \end{array}$$ (22)

where

$$\begin{array}{ccc} A & = & (\begin{array}{cc} a_{11} & a_{12}\\ a_{21} & a_{22} \end{array})=(\begin{array}{cc} \frac{1}{2}\sigma_{s}^{2}S^{2} & \frac{1}{2}\rho_{sc}\sigma_{s}\sigma_{c}S\\ \frac{1}{2}\rho_{sc}\sigma_{s}\sigma_{c}S & \frac{1}{2}\sigma_{c}^{2} \end{array}),\\ \underline{b} & = & (\begin{array}{c} b_{1}\\ b_{2} \end{array})=(\begin{array}{c} k_{s}\left(n\left(t\right)-\ln S\right)S-\lambda_{s}\sigma_{s}S-\delta S-\sigma_{s}^{2}S\\ k_{c}\left(\theta_{c}-\delta \right)-\lambda_{c}\sigma_{c}-\frac{1}{2}\rho_{sc}\sigma_{s}\sigma_{c} \end{array}), \end{array}$$ (23)

$$\begin{array}{c} \bar{c}=r+k_{s}\left(n\left(t\right)-\ln S\right)-\delta -\lambda_{s}\sigma_{s}-\sigma_{s}^{2}-k_{s}-k_{c}. \end{array}$$

Since the homogeneous Dirichlet boundary conditions are convenient for theoretical discussion, we transform Eqs (19)–(21) to be homogeneous, which is not necessary in computations. To this purpose, we introduce a new function

$$\begin{array}{c} u\left(S,\delta ,t\right)=e^{\beta t}\left(F_{0}-F\right), \end{array}$$ (24)

where $\beta =\frac{1}{2}(\sigma_{s}^{2}+\sigma_{c}^{2})$ and *F*_0_(*S*, *δ*) is a twice differentiable function satisfying the boundary conditions in Eq (20). Transforming *F* in Eq (19) into the new function *u*, we have

$$\begin{array}{c} \left\{ \begin{array}{c} 𝓛u\leq f,\\ u-u^{⋆}\leq 0,\\ \left(𝓛u-f\right)\cdot \left(u-u^{⋆}\right)=0, \end{array}\right. \end{array}$$ (25)

where

$$\begin{array}{c} \left\{ \begin{array}{c} 𝓛u=-u_{t}-\nabla \cdot \left(A\nabla u+\underline{b}u\right)+\underline{c}u,\\ \;\underline{c}=\bar{c}+\beta ,u^{⋆}=e^{\beta t}\left(F_{0}-F^{⋆}\right),f\left(S,\delta ,t\right)=e^{\beta t}LF_{0}. \end{array}\right. \end{array}$$ (26)

At the same time, the boundary and the terminal conditions in Eqs (20)–(21) are also transformed into

$$\begin{array}{c} u(0,\delta ,t)=0=u\left(S_{\max},\delta ,t\right),\;\;t\in \left[0,T\right]\;\;\text{and}\;\;\delta \in \left[\delta_{\min},\delta_{\max}\right],\\ u(S,\delta_{\min},t)=0=u\left(S,\delta_{\max},t\right),\;\;t\in \left[0,T\right]\;\;\text{and}\;\;S\in \left[0,S_{\max}\right], \end{array}$$

and *u*(*S*, *δ*, *T*) = *u* ^⋆^(*S*, *δ*), respectively.

### Formulation of the complementarity problem into a variational inequality problem

Next we reformulate Eq (25) as a variational inequality problem in an appropriate functional setting. To this end, we first introduce some standard notations. We define Ω = (0, *S*_max_) × (*δ*_min_, *δ*_max_) and Γ as the computational domain and its boundary, respectively. Let *L* ^*p*^(Ω) be the space of all *p*-integrable functions for any 1 ≤ *p* ≤ ∞ on Ω with the norm ‖⋅‖_*L*^*p*^(Ω)_. For the standard Sobolev space *H* ^*m*,*p*^(Ω), for *p* = 2 we use *H* ^*m*^(Ω) and ‖⋅‖_*m*,Ω_ to stand for *H* ^*m*,2^(Ω) and ‖⋅‖_*m*,2,Ω_, respectively. Moreover, a weighted Sobolev space $H_{\omega }^{1}(\Omega )$ and its norm ‖⋅‖_1,*ω*_ are defined as follows:

$$\begin{array}{c} H_{\omega }^{1}\left(\Omega \right)=\{v:v,Sv_{S},v_{\delta }\in L^{2}\left(\Omega \right)\},\;\;\;\;{∥v∥}_{1,\omega }^{2}=\int_{\Omega }\left(S^{2}v_{S}^{2}+v_{\delta }^{2}+v^{2}\right)d\Omega . \end{array}$$

Also, we define

$$\begin{array}{c} H_{0,\omega }^{1}\left(\Omega \right)=\{v:v\in H_{\omega }^{1}{\left(\Omega \right),\;\;v|}_{\Gamma }=0\}, \end{array}$$

$$\begin{array}{c} 𝓚=\{v\left(t\right):v\left(t\right)\in H_{0,\omega }^{1}\left(\Omega \right),v\left(t\right)\leq u^{⋆}\left(t\right),\;\;\text{a.e.}\;\;\;\text{in}\;\left(0,T\right)\}, \end{array}$$

where *u* ^⋆^(*t*) is defined by Eq (26). Clearly, 𝓚 is a convex and closed subset of $H_{0,\omega }^{1}(\Omega )$. We also define the norm of *L* ^*p*^(0, *T*; *H*(Ω)) for any Hilbert space *H*(Ω) as follows:

$$\begin{array}{c} {∥v(\cdot ,\cdot ,t)∥}_{L^{p}\left(0,T;H\left(\Omega \right)\right)}={\left(\int_{0}^{T}{∥v(\cdot ,\cdot ,t)∥}_{H}^{p}dt\right)}^{1/p}. \end{array}$$

Hereinafter, *v*(⋅, ⋅, *t*) will be written simply as *v*(*t*) when there is no confusion.

Now, we are in the position to define our variational inequality problem.

**Problem 1.** *Find* *u* ∈ 𝓚 *such that for all* *v* ∈ 𝓚,

$$\begin{array}{c} (-\frac{\partial u}{\partial t},v-u)+B\left(u,v-u;t\right)\geq \left(f,v-u\right)\;\;\text{a.e.}\;\;\text{in}\;\;\left(0,T\right), \end{array}$$ (27)

*where* *B*(⋅, ⋅; *t*) *is a bilinear form defined by*

$$\begin{array}{c} B\left(u,v;t\right)=\left(A\nabla u+\underline{b}u,\nabla v\right)+\left(\underline{c}u,v\right),\;\;u,v\;\;\in H_{0,\omega }^{1}\left(\Omega \right). \end{array}$$ (28)

For this variational inequality problem, we have the following theorem.

**Theorem 1.** *Problem 1 is the variational form of the complementarity problem* Eq (25).

**Proof.** For any *w* ∈ 𝓚, it follows from the definition of 𝓚 that

$$\begin{array}{c} w-u^{⋆}\leq 0\;\;\text{a.e.}\;\;\text{on}\;\;\Theta =\Omega \times \left(0,T\right). \end{array}$$

Multiplying both sides of the first inequality of Eq (25) by *w*−*u* ^⋆^, we obtain by integration that

$$\begin{array}{c} (-\frac{\partial u}{\partial t},w-u^{⋆})-\left(\nabla \cdot \left(A\nabla u+\underline{b}u\right)-\underline{c}u,w-u^{⋆}\right)\geq \left(f,w-u^{⋆}\right),\;\;\text{a.e.}\;\;\text{in}\;\;\left(0,T\right). \end{array}$$

Using the Gauss-divergence theory, we obtain

$$\begin{array}{c} (-\frac{\partial u}{\partial t},w-u^{⋆})+B\left(u,w-u^{⋆};t\right)\geq \left(f,w-u^{⋆}\right),\;\;\text{a.e.}\;\;\text{in}\;\;\left(0,T\right). \end{array}$$ (29)

Since 𝓚 is a convex and closed subset of $H_{0,\omega }^{1}(\Omega )$, we may write *w* as *w* = *θv* + (1−*θ*)*u*, where *u*, *v* ∈ 𝓚 and *θ* ∈ [0, 1]. Therefore, Eq (29) becomes

$$\begin{array}{ccc} & & (-\frac{\partial u}{\partial t},\theta \left(v-u\right))+B\left(u,\theta \left(v-u\right);t\right)\\ & & \;\geq \left(f,\theta \left(v-u\right)\right)-((-\frac{\partial u}{\partial t},u-u^{⋆})+B\left(u,u-u^{⋆};t\right)-\left(f,u-u^{⋆}\right)). \end{array}$$ (30)

On the other hand, from the third relationship of Eq (25) we know that

$$\begin{array}{c} (𝓛u-f,u-u^{⋆})=0, \end{array}$$

i.e.

$$\begin{array}{c} (-\frac{\partial u}{\partial t},u-u^{⋆})+B\left(u,u-u^{⋆};t\right)-\left(f,u-u^{⋆}\right)=0. \end{array}$$

Therefore, Eq (30) reduces to

$$\begin{array}{c} (-\frac{\partial u}{\partial t},\theta \left(v-u\right))+B\left(u,\theta \left(v-u\right);t\right)\geq \left(f,\theta \left(v-u\right)\right), \end{array}$$

from which we can obtain

$$\begin{array}{c} (-\frac{\partial u}{\partial t},v-u)+B\left(u,v-u;t\right)\geq \left(f,v-u\right),\;\;\text{a.e.}\;\;\text{in}\;\;\left(0,T\right). \end{array}$$

**Lemma 1.** *There exist positive constants C and M, such that for any* $v,w\in H_{0,\omega }^{1}(\Omega )$, *there hold*

$$\begin{array}{c} B\left(v,v;t\right)\geq C{∥v∥}_{1,\omega }^{2}\;\text{and}\;\left|B\left(v,w;t\right)\right|\leq M{∥v∥}_{1,\omega }{∥w∥}_{1,\omega }. \end{array}$$

**Proof.** For any $v\in H_{0,\omega }^{1}(\Omega )$, we have via integration by parts that

$$\begin{array}{c} \int_{\Omega }\underline{b}v\cdot \nabla vd\Omega =\int_{\partial \Omega }v^{2}\underline{b}\cdot nds-\int_{\Omega }v\nabla \cdot \left(\underline{b}v\right)d\Omega =-\int_{\Omega }v\underline{b}\cdot \nabla vd\Omega -\int_{\Omega }v^{2}\nabla \cdot \underline{b}d\Omega , \end{array}$$ (31)

which leads to

$$\begin{array}{c} \int_{\Omega }\underline{b}v\cdot \nabla vd\Omega =-\frac{1}{2}\int_{\Omega }\nabla \cdot \underline{b}v^{2}d\Omega . \end{array}$$

From Eqs (28) and (31) we obtain

$$\begin{array}{ccc} B(v,v;t) & = & \left(A\nabla v+\underline{b}v,\nabla v\right)+\underline{c}\left(v,v\right)\\ & = & \left(A\nabla v,\nabla v\right)+\left(\underline{b}v,\nabla v\right)+\underline{c}\left(v,v\right)\\ & = & \int_{\Omega }\left(\sigma_{s}^{2}S^{2}v_{S}^{2}+\sigma_{c}^{2}v_{\delta }^{2}\right)d\Omega +\left(\underline{c}-\frac{1}{2}\nabla \cdot \underline{b}\right)\left(v,v\right)\\ & = & \int_{\Omega }\left(\sigma_{s}^{2}S^{2}v_{S}^{2}+\sigma_{c}^{2}v_{\delta }^{2}\right)d\Omega +\left(\frac{1}{2}\left(k_{s}\left(n\left(t\right)-\ln S\right)-\lambda_{s}\sigma_{s}-y-\sigma_{s}^{2}-k_{s}-k_{c}\right)+r+\beta \right){∥v∥}_{0}^{2}\\ & \geq & C{∥v∥}_{1,\omega }^{2}. \end{array}$$

Now, let us show the continuity of *B*. For any $v,w\in H_{0,\omega }^{1}(\Omega )$, we have

$$\begin{array}{c} \left|B\left(v,w;t\right)|=\right|\left(A\nabla v+\underline{b}v,\nabla w\right)+\underline{c}\left(v,w\right)\left|\leq \right|\left(A\nabla v,\nabla w\right)\left|+\right|\left(\underline{b}v,\nabla w\right)\left|+\right|\underline{c}\left(v,w\right)|. \end{array}$$ (32)

For |(*A*∇*v*, ∇*w*)| in Eq (32), it follows from the Cauchy-Schwartz inequality that

$$\begin{array}{ccc} \left|(A\nabla v,\nabla w)\right| & = & \left|\int_{\Omega }\left(\frac{1}{2}\sigma_{s}^{2}S^{2}v_{S}w_{S}+\frac{1}{2}\sigma_{c}^{2}v_{\delta }w_{\delta }\right)d\Omega \right|\\ & \leq & \frac{1}{2}{\left(\int_{\Omega }\sigma_{s}^{2}S^{2}v_{S}^{2}d\Omega \right)}^{\frac{1}{2}}{\left(\int_{\Omega }\sigma_{s}^{2}S^{2}w_{S}^{2}d\Omega \right)}^{\frac{1}{2}}\\ & & \;+\frac{1}{2}{\left(\int_{\Omega }\sigma_{c}^{2}v_{\delta }^{2}d\Omega \right)}^{\frac{1}{2}}{\left(\int_{\Omega }\sigma_{c}^{2}w_{\delta }^{2}d\Omega \right)}^{\frac{1}{2}}\\ & \leq & M{\left(\int_{\Omega }\left(\sigma_{s}^{2}S^{2}v_{S}^{2}+\sigma_{c}^{2}v_{\delta }^{2}\right)d\Omega \right)}^{\frac{1}{2}}{\left(\int_{\Omega }\left(\sigma_{s}^{2}S^{2}w_{S}^{2}+\sigma_{c}^{2}w_{\delta }^{2}\right)d\Omega \right)}^{\frac{1}{2}}\\ & \leq & M{∥v∥}_{1,\omega }{∥w∥}_{1,\omega }. \end{array}$$

For $\left|(\underline{b}v,\nabla w)\right|$ in Eq (32), it follows from the expression of $\underline{b}$ in Eq (23) and the Cauchy-Schwarz inequality that

$$\begin{array}{ccc} \left|(\underline{b}v,\nabla w)\right| & = & \left|\int_{\Omega }\underline{b}v\cdot \nabla wd\Omega |=|\int_{\partial \Omega }vw\underline{b}\cdot nds-\int_{\Omega }w\nabla \cdot \left(\underline{b}v\right)d\Omega \right|\\ & = & \left|-\int_{\Omega }w\underline{b}\cdot \nabla vd\Omega -\int_{\Omega }vw\nabla \cdot \underline{b}d\Omega \right|\\ & \leq & \left|\int_{\Omega }w\underline{b}\cdot \nabla vd\Omega |+|\int_{\Omega }vw\nabla \cdot \underline{b}d\Omega \right|\\ & \leq & \left|\int_{\Omega }w\left(b_{1}v_{S}+b_{2}v_{\delta }\right)d\Omega |+|\int_{\Omega }vw\nabla \cdot \underline{b}d\Omega \right|\\ & \leq & M{∥w∥}_{0}{∥v∥}_{1,\omega }+M{∥v∥}_{0}{∥w∥}_{0}. \end{array}$$

For $\left|\underline{c}(v,w)\right|$ in Eq (32), it is easy to see

$$\begin{array}{c} |\underline{c}\left(v,w\right)|\leq M{∥v∥}_{0}{∥w∥}_{0}. \end{array}$$

Summarizing the above, the continuity of *B* is obtained as follows:

$$\begin{array}{c} B\left(v,w;t\right)\leq M\left({∥v∥}_{1,\omega }{∥w∥}_{1,\omega }+{∥w∥}_{0}{∥v∥}_{1,\omega }+{∥v∥}_{0}{∥w∥}_{0}\right)\leq M{∥v∥}_{1,\omega }{∥w∥}_{1,\omega }. \end{array}$$

**Remark 3.** *From Lemma 1 we know that* ${\left\|\cdot \right\|}_{B}=\sqrt{B(\cdot ,\cdot )}$ *is a norm*.

Lemma 1, together with the theory of abstract variational inequalities, yields the following theorem.

**Theorem 2.** *Problem 1 has a unique solution*.

### The power penalty approach

First of all, we introduce the following nonlinear variational inequality to obtain the power penalty method:

Seek $u_{\lambda }\in H_{0,\omega }^{1}(\Omega )$ to satisfy that for all $v\in H_{0,\omega }^{1}(\Omega )$,

$$\begin{array}{c} (-\frac{\partial u_{\lambda }}{\partial t},v-u_{\lambda })+B\left(u_{\lambda },v-u_{\lambda };t\right)+j\left(v\right)-j\left(u_{\lambda }\right)\geq \left(f,v-u_{\lambda }\right)\;\;\text{a.e.}\;\;\text{in}\;\;\left(0,T\right), \end{array}$$ (33)

where

$$\begin{array}{c} j\left(v\right)=\frac{\lambda k}{k+1}{\left[v-u^{⋆}\right]}_{+}^{\frac{k+1}{k}},\;\;k>0,\;\;\lambda >1, \end{array}$$ (34)

and [*z*]_+_ = max{0, *z*}. According to Lemma 1 and the lower semi-continuity of *j*, the problem has a unique solution. In addition, *j*(*v*) is differentiable, which means that Eq (33) is equivalent to the following problem.

**Problem 2.** *Seek* $u_{\lambda }\in H_{0,\omega }^{1}(\Omega )$ *to satisfy that for all* $v\in H_{0,\omega }^{1}(\Omega )$,

$$\begin{array}{c} (-\frac{\partial u_{\lambda }}{\partial t},v)+B\left(u_{\lambda },v;t\right)+\left(j^{\prime }\left(u_{\lambda }\right),v\right)=\left(f,v\right)\;\;\text{a.e.}\;\;\text{in}\;\;\left(0,T\right), \end{array}$$ (35)

*where*

$$\begin{array}{c} j^{\prime }\left(v\right)=\lambda {\left[v-u^{⋆}\right]}_{+}^{\frac{1}{k}}. \end{array}$$ (36)

Note that the penalized variational equation of variational inequality Eq (27) is just Eqs (35)–(36), and the penalized equation, which approximates Eq (25), can be written as:

$$\begin{array}{c} 𝓛u_{\lambda }+\lambda {\left[u_{\lambda }-u^{⋆}\right]}_{+}^{\frac{1}{k}}=f,\;\;\left(S,\delta ,t\right)\in \Omega \times \left[0,T\right), \end{array}$$ (37)

with the following boundary and final conditions:

$$\begin{array}{c} u_{\lambda }{\left(S,\delta ,t\right)|}_{\Gamma }=0\;\;\text{and}\;\;u_{\lambda }\left(S,\delta ,T\right)=u^{⋆}\left(S,\delta \right). \end{array}$$ (38)

It has been proved in several papers [37–40] that the solution of Eq (37) converges to that of Eq (19) at the rate 𝓞(*λ* ^−*k*/2^) in a Sobolev norm as *λ* → +∞.

## The fitted finite volume method

As the valuation models for futures and European options are the special forms of Eq (37), the so-called fitted finite volume method is presented only for the American option model here, and it can be also applied to the other two models. The innovation of this method is that it combines two existing techniques, a finite volume method and a fitted approximation, together, in which the flux of a given function is approximated by a constant locally, yielding a locally nonlinear approximation to the function. In what follows, we will develop the method for our two-dimensional nonlinear partial differential equation with a penalty term.

### Boundary conditions

First of all, how to determine the boundary condition functions *g*_1_(*S*, *t*) and *g*_2_(*S*, *t*) is discussed below.

1. The boundary condition *g*_1_(*S*, *t*) on the boundary *δ* = *δ*_min_ is determined by solving the following parabolic partial differential equation:
  $$\begin{array}{c} \left\{ \begin{array}{c} -\frac{\partial F}{\partial t}-\frac{1}{2}\sigma_{s}^{2}S^{2}\frac{\partial^{2}F}{\partial S^{2}}-h\left(S,\delta_{\min},t\right)\frac{\partial F}{\partial S}+rF-\lambda {\left[F^{*}-F\right]}_{+}^{\frac{1}{k}}=0,\\ \;F\left(0,t\right)=0,F\left(S_{\max},t\right)=S_{\max}-K,\\ \;F(S,T)=\max\;(S-K,0). \end{array}\right. \end{array}$$ (39)
2. The boundary condition *g*_2_(*S*, *t*) on the boundary *δ* = *δ*_max_ is determined by solving the following initial-boundary problem:
  $$\begin{array}{c} \left\{ \begin{array}{c} -\frac{\partial F}{\partial t}-\frac{1}{2}\sigma_{s}^{2}S^{2}\frac{\partial^{2}F}{\partial S^{2}}-h\left(S,\delta_{\max},t\right)\frac{\partial F}{\partial S}+rF-\lambda {\left[F^{*}-F\right]}_{+}^{\frac{1}{k}}=0,\\ \;F\left(0,t\right)=0,F\left(S_{\max},t\right)=S_{\max}-K,\\ \;F(S,T)=\max\;(S-K,0). \end{array}\right. \end{array}$$ (40)

### The fitted finite volume method

From Eq (24) we can easily find that the problem Eqs (37)–(38) can be rewritten as

$$\begin{array}{c} -F_{t}-\nabla \cdot \left(A\nabla F+\underline{b}F\right)+\bar{c}F-\lambda {\left[F^{*}-F\right]}_{+}^{\frac{1}{k}}=0,\;\;\;\;\left(S,\delta ,t\right)\in \Omega \times \left[0,T\right) \end{array}$$ (41)

with the boundary and the terminal conditions Eqs (20)–(21). In what follows, we let *I*_*S*_ = (0, *S*_max_) and *I*_*δ*_ = (*δ*_min_, *δ*_max_), and divide the intervals *I*_*S*_ and *I*_*δ*_ into *N*_*S*_ and *N*_*δ*_ sub-intervals, respectively:

$$\begin{array}{c} I_{S_{i}}≔\left(S_{i},S_{i+1}\right),\;\;I_{\delta_{j}}≔\left(\delta_{j},\delta_{j+1}\right),\;\;\;\;i=0,1,\cdots ,N_{S}-1,\;\;j=0,1,\cdots ,N_{\delta }-1, \end{array}$$

in which

$$\begin{array}{c} 0=S_{0}<S_{1}<\cdots <S_{N_{S}}=S_{\max}\;\;\;\;\text{and}\;\;\;\;\delta_{\min}=\delta_{0}<\delta_{1}<\cdots <\delta_{N_{\delta }}=\delta_{\max}. \end{array}$$

This defines a mesh on *I*_*S*_ × *I*_*δ*_ with all mesh lines perpendicular to one of the axes.

Next we define another partition of *I*_*S*_ × *I*_*δ*_ by letting

$$\begin{array}{c} S_{i-\frac{1}{2}}=\frac{S_{i-1}+S_{i}}{2},\;S_{i+\frac{1}{2}}=\frac{S_{i}+S_{i+1}}{2},\;\delta_{j-\frac{1}{2}}=\frac{\delta_{j-1}+\delta_{j}}{2},\;\delta_{j+\frac{1}{2}}=\frac{\delta_{j}+\delta_{j+1}}{2} \end{array}$$

for *i* = 1, 2, ⋯, *N*_*S*_−1 and *j* = 1, 2, ⋯, *N*_*δ*_−1, and $S_{-\frac{1}{2}}=0$, $S_{N_{S}+\frac{1}{2}}=S_{\max}$, $\delta_{-\frac{1}{2}}=\delta_{\min}$, and $\delta_{N_{\delta }+\frac{1}{2}}=\delta_{\max}$. Also, we put $h_{S_{i}}=S_{i+\frac{1}{2}}-S_{i-\frac{1}{2}}$ and $h_{\delta_{j}}=\delta_{j+\frac{1}{2}}-\delta_{j-\frac{1}{2}}$ for each *i* = 0, 1, ⋯, *N*_*S*_ and *j* = 0, 1, ⋯, *N*_*δ*_.

Integrate Eq (41) over ${ℛ}_{i,j}=[S_{i-\frac{1}{2}},S_{i+\frac{1}{2}}]\times [\delta_{j-\frac{1}{2}},\delta_{j+\frac{1}{2}}]$ to obtain

$$\begin{array}{ccc} & & -\int_{S_{i-\frac{1}{2}}}^{S_{i+\frac{1}{2}}}\int_{\delta_{j-\frac{1}{2}}}^{\delta_{j+\frac{1}{2}}}\frac{\partial F}{\partial t}dSd\delta -\int_{S_{i-\frac{1}{2}}}^{S_{i+\frac{1}{2}}}\int_{\delta_{j-\frac{1}{2}}}^{\delta_{j+\frac{1}{2}}}\nabla \cdot \left(A\nabla F+\underline{b}F\right)dSd\delta \\ \; & & +\int_{S_{i-\frac{1}{2}}}^{S_{i+\frac{1}{2}}}\int_{\delta_{j-\frac{1}{2}}}^{\delta_{j+\frac{1}{2}}}\bar{c}FdSd\delta -\lambda \int_{S_{i-\frac{1}{2}}}^{S_{i+\frac{1}{2}}}\int_{\delta_{j-\frac{1}{2}}}^{\delta_{j+\frac{1}{2}}}{\left[F^{*}-F\right]}_{+}^{\frac{1}{k}}dSd\delta =0, \end{array}$$

for *i* = 1, 2, ⋯, *N*_*S*_−1, *j* = 1, 2, ⋯, *N*_*δ*_−1. Applying the mid-point quadrature rule to the above equation, we have

$$\begin{array}{c} -\frac{\partial F_{i,j}}{\partial t}R_{i,j}-\int_{{𝓡}_{i,j}}\nabla \cdot \left(A\nabla F+\underline{b}F\right)dSd\delta +{\bar{c}}_{i,j}F_{i,j}R_{i,j}-\lambda {\left[F_{i,j}^{*}-F_{i,j}\right]}_{+}^{\frac{1}{k}}R_{i,j}=0 \end{array}$$ (42)

for *i* = 1, 2, ⋯, *N*_*S*_−1, *j* = 1, 2, ⋯, *N*_*δ*_−1, where $R_{i,j}=(S_{i+\frac{1}{2}}-S_{i-\frac{1}{2}})\times (\delta_{j+\frac{1}{2}}-\delta_{j-\frac{1}{2}})$, ${\bar{c}}_{i,j}=\bar{c}(S_{i},\delta_{j},t)$, *F*_*i*,*j*_ = *F*(*S*_*i*_, *δ*_*j*_, *t*), and $F_{i,j}^{*}=F^{*}(S_{i},\delta_{j},t).$

Now we consider how to approximate the second term in Eq (42). First of all, it follows from the definition of flux $A\nabla F+\underline{b}F$ and integration by parts that

$$\begin{array}{ccc} \int_{{𝓡}_{i,j}}\nabla \cdot \left(A\nabla F+\underline{b}F\right)dSd\delta & = & \int_{\partial {𝓡}_{i,j}}\left(A\nabla F+\underline{b}F\right)\cdot lds\\ & = & \int_{\left(S_{i+\frac{1}{2}},\delta_{j-\frac{1}{2}}\right)}^{\left(S_{i+\frac{1}{2}},\delta_{j+\frac{1}{2}}\right)}\left(a_{11}F_{S}+a_{12}F_{\delta }+b_{1}F\right)d\delta \\ & - & \int_{\left(S_{i-\frac{1}{2}},\delta_{j-\frac{1}{2}}\right)}^{\left(S_{i-\frac{1}{2}},\delta_{j+\frac{1}{2}}\right)}\left(a_{11}F_{S}+a_{12}F_{\delta }+b_{1}F\right)d\delta \\ & + & \int_{\left(S_{i-\frac{1}{2}},\delta_{j+\frac{1}{2}}\right)}^{\left(S_{i+\frac{1}{2}},\delta_{j+\frac{1}{2}}\right)}\left(a_{21}F_{S}+a_{22}F_{\delta }+b_{2}F\right)dS\\ & - & \int_{\left(S_{i+\frac{1}{2}},\delta_{j-\frac{1}{2}}\right)}^{\left(S_{i-\frac{1}{2}},\delta_{j-\frac{1}{2}}\right)}\left(a_{21}F_{S}+a_{22}F_{\delta }+b_{2}F\right)dS, \end{array}$$ (43)

where *l* denote the unit vector outward-normal to ∂ℛ_*i*,*j*_.

Next we deal with Eq (43) term by term. In fact, for the first term we can approximate the integral by a constant, i.e,

$$\begin{array}{c} \int_{\left(S_{i+\frac{1}{2}},\delta_{j-\frac{1}{2}}\right)}^{\left(S_{i+\frac{1}{2}},\delta_{j+\frac{1}{2}}\right)}\left(a_{11}F_{S}+a_{12}F_{\delta }+b_{1}F\right)d\delta \approx \left(a_{11}F_{S}+a_{12}F_{\delta }+b_{1}F\right){|}_{\left(S_{i+\frac{1}{2}},\delta_{j}\right)}\cdot h_{\delta_{j}}. \end{array}$$

Clearly, we now need to derive approximations of the $(A\nabla F+\underline{b}F)\cdot l$ defined above at the mid-point, $\left(S_{i+\frac{1}{2}},\delta_{j}\right)$, on the interval *I*_*S*_*i*__ for any *i* = 0, 1, ⋯, *N*_*S*_−1. Next we discuss it in detail.

**Case 1:** For *i* ≥ 1.

According to Eq (23) we have

$$\begin{array}{ccc} a_{11}F_{S}+a_{12}F_{\delta }+b_{1}F & = & \frac{1}{2}\sigma_{s}^{2}S^{2}F_{S}+\frac{1}{2}\rho_{sc}\sigma_{s}\sigma_{c}SF_{\delta }+S\left(k_{s}\left(n\left(t\right)-\ln S\right)-\delta -\lambda_{s}\sigma_{s}-\sigma_{s}^{2}\right)F\\ & = & S(\frac{1}{2}\sigma_{s}^{2}SF_{S}+\frac{1}{2}\rho_{sc}\sigma_{s}\sigma_{c}F_{\delta }+\left(k_{s}\left(n\left(t\right)-\ln S\right)-\delta -\lambda_{s}\sigma_{s}-\sigma_{s}^{2}\right)F). \end{array}$$ (44)

Following the idea in [29], we approximate the term *a*_11_ *F*_*S*_+*b*_1_ *F* by solving the following two-point boundary value problem:

$$\begin{array}{c} {\left(\frac{1}{2}\sigma_{s}^{2}SF_{S}+\left(k_{s}\left(n\left(t\right)-\ln S\right)-\delta -\lambda_{s}\sigma_{s}-\sigma_{s}^{2}\right)F\right)}^{\prime }\equiv {\left(aSF_{S}+b_{i+\frac{1}{2},j}F\right)}^{\prime }=0, \end{array}$$ (45a)

$$\begin{array}{c} F\left(S_{i},\delta_{j}\right)=F_{i,j},\;F\left(S_{i+1},\delta_{j}\right)=F_{i+1,j}, \end{array}$$ (45b)

where $a=\frac{1}{2}\sigma_{s}^{2}$ and $b=k_{s}(n(t)-\ln S)-\delta -\lambda_{s}\sigma_{s}-\sigma_{s}^{2}$, $b_{i+\frac{1}{2},j}=b(S_{i+\frac{1}{2}},\delta_{j})$, and *F*_*i*,*j*_ and *F*_*i*+1,*j*_ denote the values of *F* at (*S*_*i*_, *δ*_*j*_) and (*S*_*i*+1_, *δ*_*j*_), respectively. Evidently, from Eq (45a) we can obtain

$$\begin{array}{c} aSF_{S}+b_{i+\frac{1}{2},j}F=C_{1}, \end{array}$$

where *C*_1_ is an arbitrary constant and can be determined by the boundary condition Eq (45b) as follows [29, 41]:

$$\begin{array}{c} C_{1}=b_{i+\frac{1}{2},j}\frac{S_{i+1}^{\alpha_{i,j}}F_{i+1,j}-S_{i}^{\alpha_{i,j}}F_{i,j}}{S_{i+1}^{\alpha_{i,j}}-S_{i}^{\alpha_{i,j}}}, \end{array}$$

where $\alpha_{i,j}=\frac{b_{i+\frac{1}{2},j}}{a}$. Then,

$$\begin{array}{c} a_{11}F_{S}+a_{12}F_{\delta }+b_{1}F\approx S_{i+\frac{1}{2}}(b_{i+\frac{1}{2},j}\frac{S_{i+1}^{\alpha_{i,j}}F_{i+1,j}-S_{i}^{\alpha_{i,j}}F_{i,j}}{S_{i+1}^{\alpha_{i,j}}-S_{i}^{\alpha_{i,j}}}+d\cdot F_{\delta }), \end{array}$$ (46)

where $d=\frac{1}{2}\rho_{sc}\sigma_{s}\sigma_{c}$. Additionally, the derivative *F*_*δ*_ can be approximated by a forward difference

$$\begin{array}{c} \frac{F_{i,j+1}-F_{i,j}}{h_{\delta_{j}}}. \end{array}$$

Then, we have

$$\begin{array}{c} \left(a_{11}F_{S}+a_{12}F_{\delta }+b_{1}F\right){|}_{\left(S_{i+\frac{1}{2}},\delta_{j}\right)}\cdot h_{\delta_{j}}\approx S_{i+\frac{1}{2}}(b_{i+\frac{1}{2},j}\frac{S_{i+1}^{\alpha_{i,j}}F_{i+1,j}-S_{i}^{\alpha_{i,j}}F_{i,j}}{S_{i+1}^{\alpha_{i,j}}-S_{i}^{\alpha_{i,j}}}+d_{i,j}\frac{F_{i,j+1}-F_{i,j}}{h_{\delta_{j}}})\cdot h_{\delta_{j}}, \end{array}$$ (47)

where *d*_*i*,*j*_ = *d*(*S*_*i*_, *δ*_*j*_). In the same way, we can also approximate the second term in Eq (43) as follows:

$$\begin{array}{c} \left(a_{11}F_{S}+a_{12}F_{\delta }+b_{1}F\right){|}_{\left(S_{i-\frac{1}{2}},\delta_{j}\right)}\cdot h_{\delta_{j}}\approx S_{i-\frac{1}{2}}(b_{i-\frac{1}{2},j}\frac{S_{i}^{\alpha_{i-1,j}}F_{i,j}-S_{i-1}^{\alpha_{i-1,j}}F_{i-1,j}}{S_{i}^{\alpha_{i-1,j}}-S_{i-1}^{\alpha_{i-1,j}}}+d_{i,j}\frac{F_{i,j+1}-F_{i,j}}{h_{\delta_{j}}})\cdot h_{\delta_{j}}. \end{array}$$ (48)

**Case 2:** For *i* = 0.

As the Eq (45a) is degenerate on (0, *S*_1_), we should rethink the problem Eqs (45a)–(45b) on (0, *S*_1_) below:

$$\begin{array}{c} {\left(aSF_{S}+b_{\frac{1}{2},j}F\right)}^{\prime }\equiv C_{2}, \end{array}$$ (49a)

$$\begin{array}{c} F\left(0,\delta_{j}\right)=F_{0,j},\;F\left(S_{1},\delta_{j}\right)=F_{1,j}, \end{array}$$ (49b)

where $b_{\frac{1}{2},j}=b(S_{\frac{1}{2}},\delta_{j})$ and *C*_2_ is an unknown constant to be determined next. After integrating both sides of above equation, we can obtain

$$\begin{array}{c} aSF_{S}+b_{\frac{1}{2},j}F=C_{2}S+C_{3}. \end{array}$$

Thus, we have

$$\begin{array}{c} \left(a_{11}F_{S}+a_{12}F_{\delta }+b_{1}F\right){|}_{\left(S_{\frac{1}{2}},\delta_{j}\right)}\cdot h_{\delta_{j}}\approx \{\frac{1}{2}\left[\left(a+b_{\frac{1}{2},j}\right)F_{1,j}-\left(a-b_{\frac{1}{2},j}\right)F_{0,j}\right]+d_{1,j}\frac{F_{1,j+1}-F_{1,j}}{h_{\delta_{j}}}\}\cdot h_{\delta_{j}}. \end{array}$$ (50)

In the approximations of last two terms of Eq (43), we do not need to consider the case 2 as before, since *δ*_0_ ≠ 0. We apply the similar method to the above and get the following results:

$$\begin{array}{c} \left(a_{21}F_{S}+a_{22}F_{\delta }+b_{2}F\right){|}_{\left(S_{i},\delta_{j+\frac{1}{2}}\right)}\cdot h_{S_{i}}\approx ({\bar{b}}_{i,j+\frac{1}{2}}\frac{e^{{\bar{\alpha }}_{i,j}\delta_{j+1}}F_{i,j+1}-e^{{\bar{\alpha }}_{i,j}\delta_{j}}F_{i,j}}{e^{{\bar{\alpha }}_{i,j}\delta_{j+1}}-e^{{\bar{\alpha }}_{i,j}\delta_{j}}}+{\bar{d}}_{i,j}\frac{F_{i+1,j}-F_{i,j}}{h_{S_{i}}})\cdot h_{S_{i}}, \end{array}$$ (51)

$$\begin{array}{c} \left(a_{21}F_{S}+a_{22}F_{\delta }+b_{2}F\right){|}_{\left(S_{i},\delta_{j-\frac{1}{2}}\right)}\cdot h_{S_{i}}\approx ({\bar{b}}_{i,j-\frac{1}{2}}\frac{e^{{\bar{\alpha }}_{i,j-1}\delta_{j}}F_{i,j}-e^{{\bar{\alpha }}_{i,j-1}\delta_{j-1}}F_{i,j-1}}{e^{{\bar{\alpha }}_{i,j-1}\delta_{j}}-e^{{\bar{\alpha }}_{i,j-1}\delta_{j-1}}}+{\bar{d}}_{i,j}\frac{F_{i+1,j}-F_{i,j}}{h_{S_{i}}})\cdot h_{S_{i}}, \end{array}$$ (52)

for *j* = 0, 1, ⋯, *N*_*δ*_−1, where ${\bar{\alpha }}_{i,j}=\frac{{\bar{b}}_{i,j+\frac{1}{2}}}{{\bar{a}}_{j}}$, $\bar{a}=\frac{1}{2}\sigma_{c}^{2}$, $\bar{b}=k_{c}(\theta_{c}-\delta )-\lambda_{c}\sigma_{c}-\frac{1}{2}\rho_{sc}\sigma_{s}\sigma_{c}$, and ${\bar{d}}_{i,j}=\frac{1}{2}\rho_{sc}\sigma_{s}\sigma_{c}S_{i}$. Hence, we obtain the following equations by combining Eqs (43), (47), and (48) with Eqs (50)–(52):

$$\begin{array}{ccc} & & -\frac{\partial F_{i,j}}{\partial t}R_{i,j}+e_{i-1,j}^{i,j}F_{i-1,j}+e_{i,j-1}^{i,j}F_{i,j-1}+e_{i,j}^{i,j}F_{i,j}+e_{i,j+1}^{i,j}F_{i,j+1}\\ & & +e_{i+1,j}^{i,j}F_{i+1,j}-\lambda {\left[F_{i,j}^{*}-F_{i,j}\right]}_{+}^{\frac{1}{k}}R_{i,j}=0, \end{array}$$ (53)

for *i* = 1, 2, ⋯, *N*_*S*_−1 and *j* = 1, 2, ⋯, *N*_*δ*_−1, where

$$\begin{array}{c} e_{0,j}^{1,j}=-\frac{1}{2}S_{\frac{1}{2}}h_{\delta_{j}}\left(a-b_{\frac{1}{2},j}\right),\;e_{1,j-1}^{1,j}=-\frac{{\bar{b}}_{1,j-\frac{1}{2}}e^{{\bar{\alpha }}_{1,j-1}\delta_{j-1}}h_{S_{1}}}{e^{{\bar{\alpha }}_{1,j-1}\delta_{j}}-e^{{\bar{\alpha }}_{1,j-1}\delta_{j-1}}}, \end{array}$$ (54)

$$\begin{array}{ccc} e_{1,j}^{1,j} & = & h_{S_{1}}(\frac{{\bar{b}}_{1,j+\frac{1}{2}}e^{{\bar{\alpha }}_{1,j}\delta_{j}}}{e^{{\bar{\alpha }}_{1,j}\delta_{j+1}}-e^{{\bar{\alpha }}_{1,j}\delta_{j}}}+\frac{{\bar{b}}_{1,j-\frac{1}{2}}e^{{\bar{\alpha }}_{1,j-1}\delta_{j}}}{e^{{\bar{\alpha }}_{1,j-1}\delta_{j}}-e^{{\bar{\alpha }}_{1,j-1}\delta_{j-1}}}+d_{1,j})\\ & & +S_{\frac{3}{2}}h_{\delta_{j}}\frac{b_{\frac{3}{2},j}S_{1}^{\alpha_{1,j}}}{S_{2}^{\alpha_{1,j}}-S_{1}^{\alpha_{1,j}}}+\frac{1}{2}S_{\frac{1}{2}}h_{\delta_{j}}\left(a+b_{\frac{1}{2},j}\right)+{\bar{c}}_{1,j}R_{1,j}, \end{array}$$ (55)

$$\begin{array}{c} e_{1,j+1}^{1,j}=-h_{S_{1}}(\frac{{\bar{b}}_{1,j+\frac{1}{2}}e^{{\bar{\alpha }}_{1,j}\delta_{j+1}}}{e^{{\bar{\alpha }}_{1,j}\delta_{j+1}}-e^{{\bar{\alpha }}_{1,j}\delta_{j}}}+d_{1,j}),\;e_{2,j}^{1,j}=-S_{\frac{3}{2}}b_{\frac{3}{2},j}\frac{S_{2}^{\alpha_{1,j}}h_{\delta_{j}}}{S_{2}^{\alpha_{1,j}}-S_{1}^{\alpha_{1,j}}}, \end{array}$$ (56)

for *j* = 1, 2, ⋯, *N*_*δ*_−1, and

$$\begin{array}{c} e_{i-1,j}^{i,j}=-S_{i-\frac{1}{2}}h_{\delta_{j}}\frac{b_{i-\frac{1}{2},j}S_{i-1}^{\alpha_{i-1,j}}}{S_{i}^{\alpha_{i-1,j}}-S_{i-1}^{\alpha_{i-1,j}}},\;e_{i,j-1}^{i,j}=-\frac{{\bar{b}}_{i,j-\frac{1}{2}}e^{{\bar{\alpha }}_{i,j-1}\delta_{j-1}}h_{S_{i}}}{e^{{\bar{\alpha }}_{i,j-1}\delta_{j}}-e^{{\bar{\alpha }}_{i,j-1}\delta_{j-1}}}, \end{array}$$ (57)

$$\begin{array}{ccc} e_{1,j}^{1,j} & = & h_{S_{i}}(\frac{{\bar{b}}_{i,j+\frac{1}{2}}e^{{\bar{\alpha }}_{i,j}\delta_{j}}}{e^{{\bar{\alpha }}_{i,j}\delta_{j+1}}-e^{{\bar{\alpha }}_{i,j}\delta_{j}}}+\frac{{\bar{b}}_{i,j-\frac{1}{2}}e^{{\bar{\alpha }}_{i,j-1}\delta_{j}}}{e^{{\bar{\alpha }}_{i,j-1}\delta_{j}}-e^{{\bar{\alpha }}_{i,j-1}\delta_{j-1}}}+d_{i,j})\\ & & +h_{\delta_{j}}(S_{i+\frac{1}{2}}\frac{b_{i+\frac{1}{2},j}S_{i}^{\alpha_{i,j}}}{S_{i+1}^{\alpha_{i,j}}-S_{i}^{\alpha_{i,j}}}+S_{i-\frac{1}{2}}\frac{b_{i-\frac{1}{2},j}S_{i}^{\alpha_{i-1,j}}}{S_{i}^{\alpha_{i-1,j}}-S_{i-1}^{\alpha_{i-1,j}}})+{\bar{c}}_{i,j}R_{i,j}, \end{array}$$ (58)

$$\begin{array}{c} e_{i,j+1}^{i,j}=-h_{S_{i}}(\frac{{\bar{b}}_{i,j+\frac{1}{2}}e^{{\bar{\alpha }}_{i,j}\delta_{j+1}}}{e^{{\bar{\alpha }}_{i,j}\delta_{j+1}}-e^{{\bar{\alpha }}_{i,j}\delta_{j}}}+d_{i,j}),\;e_{i+1,j}^{i,j}=-S_{i+\frac{1}{2}}b_{i+\frac{1}{2},j}\frac{S_{i+1}^{\alpha_{i,j}}h_{\delta_{j}}}{S_{i+1}^{\alpha_{i,j}}-S_{i}^{\alpha_{i,j}}}, \end{array}$$ (59)

for *i* = 2, 3, ⋯, *N*_*S*_−1, *j* = 1, 2, ⋯, *N*_*δ*_−1, and $e_{m,n}^{i,j}=0$ if *m* ≠ *i*−1, *i*, *i*+1 and *n* ≠ *j*−1, *j*, *j*+1. It can be easily seen that Eq (53) is an (*N*_*S*_−1)^2^ × (*N*_*δ*_−1)^2^ linear system of equations for

$$\begin{array}{c} F={\left(F_{1,1},\cdots ,F_{1,N_{\delta }-1},F_{2,1},\cdots ,F_{2,N_{\delta }-1},\cdots ,F_{N_{S}-1,1},F_{N_{S}-1,2},\cdots ,F_{N_{S}-1,N_{\delta }-1}\right)}^{⊤}. \end{array}$$

Note that for *i* = 1, 2, ⋯, *N*_*S*_ and *j* = 1, 2, ⋯, *N*_*δ*_, *F*_0, *j*_(*t*), *F*_*i*,0_(*t*), *F*_0, *N*_*δ*__(*t*) and *F*_*N*_*S*_,0_(*t*) are equal to the given boundary conditions. Obviously, the coefficient matrix of Eq (53) is penta-diagonal.

Let

$$\begin{array}{c} E_{i,j}=\left(0,\cdots ,0,e_{i-1,j}^{i,j},0,\cdots ,0,e_{i,j-1}^{i,j},e_{i,j}^{i,j},e_{i,j+1}^{i,j},0,\cdots ,0,e_{i+1,j}^{i,j},0,\cdots ,0\right) \end{array}$$

for *i* = 1, 2, ⋯, *N*_*S*_−1 and *j* = 1, 2, ⋯, *N*_*δ*_−1. Now we are in the position to discuss the time-discretization of the system Eq (53). To this purpose, we first rewrite Eq (53) as

$$\begin{array}{c} -\frac{\partial F_{i,j}}{\partial t}R_{i,j}+E_{i,j}F+p\left(F_{i,j}\right)=0, \end{array}$$ (60)

where

$$\begin{array}{c} p\left(F_{i,j}\right)=-\lambda R_{i,j}{\left[F_{i,j}^{*}-F_{i,j}\right]}_{+}^{\frac{1}{k}}. \end{array}$$ (61)

Then, we select *M*−1 points numbered from *t*_1_ to *t*_*M*−1_ between 0 and *T* and let *T* = *t*_0_, *t*_*M*_ = 0 to form a partition: *T* = *t*_0_ > *t*_1_ > ⋯ > *t*_*M*_ = 0. Thus, the full discrete form of Eq (60) can be obtained by applying the two-level implicit time-stepping method with a splitting parameter $\theta \in [\frac{1}{2},1]$ to it as follows:

$$\begin{array}{c} \left(\theta E^{m+1}+G^{m}\right)F^{m+1}+\theta D\left(F^{m+1}\right)=\left(G^{m}-\left(1-\theta \right)E^{m}\right)F^{m}-\left(1-\theta \right)D\left(F^{m}\right), \end{array}$$ (62)

where

$$\begin{array}{c} F^{m}={\left(F_{1,1}^{m},\cdots ,F_{1,N_{\delta }-1}^{m},F_{2,1}^{m},\cdots ,F_{2,N_{\delta }-1}^{m},\cdots ,F_{N_{S}-1,1}^{m},\cdots ,F_{N_{S}-1,N_{\delta }-1}^{m}\right)}^{⊤},\\ E^{m}={\left(E_{1,1}^{m},\cdots ,E_{1,N_{\delta }-1}^{m},E_{2,1}^{m},\cdots ,E_{2,N_{\delta }-1}^{m},\cdots ,E_{N_{S}-1,1}^{m},\cdots ,E_{N_{S}-1,N_{\delta }-1}^{m}\right)}^{⊤},\\ G^{m}=\text{diag}\;{\left(-R_{1,1}/\Delta t_{m},\cdots ,-R_{N_{S}-1,N_{\delta }-1}/\Delta t_{m}\right)}^{⊤},\\ D(F^{m})={\left(p\left(F_{1,1}^{m}\right),\cdots ,p\left(F_{N_{S}-1,N_{\delta }-1}^{m}\right)\right)}^{⊤}, \end{array}$$ (63)

for *m* = 0, 1, ⋯, *M*−1. Note that Δ*t*_*m*_ = *t*_*m*+1_−*t*_*m*_ < 0, $E_{i,j}^{m}=E_{i,j}(t_{m})$, and *F* ^*m*^ denotes the approximation of *F* at *t* = *t*_*m*_.

### The solution of the discrete system

The standard Newton method is employed to solve the nonlinear discrete system Eq (62). Eq (61) clearly indicates that $p^{\prime }(V_{i,j}^{m})\to \infty$ as $F_{i,j}^{*}-F_{i,j}\to 0^{+}$ when *k* > 1. So, we need to smooth out $p(F_{i,j}^{m})$ by redefining it as follows:

$$\begin{array}{c} p\left(F_{i,j}^{m}\right)=\{\begin{array}{cc} -\lambda R_{i,j}{\left[F_{i,j}^{*}-F_{i,j}^{m}\right]}_{+}^{\frac{1}{k}}, & F_{i,j}^{*}-F_{i,j}^{m}\geq \epsilon ,\\ -\lambda R_{i,j}(\epsilon^{\frac{1}{k}-n+1}\left(n-\frac{1}{k}\right){\left[F_{i,j}^{*}-F_{i,j}^{m}\right]}_{+}^{n-1} & \\ +\epsilon^{\frac{1}{k}-n}\left(\frac{1}{k}-n+1\right){\left[F_{i,j}^{*}-F_{i,j}^{m}\right]}_{+}^{n}), & F_{i,j}^{*}-F_{i,j}^{m}<\epsilon , \end{array} \end{array}$$ (64)

where *k* > 0, *n* is a positive integer, and 0 < *ϵ* ≪ 1 is a transition parameter. Applying Newton method to Eq (62), we can get

$$\begin{array}{ccc} \left[\theta E^{m+1}+G^{m}+\theta J_{D}\left(\omega^{q-1}\right)\right]d\omega^{q} & = & \left[G^{m}-\left(1-\theta \right)E^{m}\right]F^{m}-\left(1-\theta \right)D\left(F^{m}\right)\\ & & \;-\left(\theta E^{m+1}+G^{m}\right)\omega^{q-1}-\theta D\left(\omega^{q-1}\right),\\ \omega^{q}=\omega^{q-1}+\gamma \cdot d\omega^{q}, \end{array}$$ (65)

for *q* = 1, 2, ⋯, where *ω* ^0^ is a given initial guess, *J*_*D*_(*ω*) denotes the Jacobian matrix of the column vector *D*(*ω*), and *γ* ∈ (0, 1] is a damping parameter used to accelerate convergence. Then, we choose

$$\begin{array}{c} F^{m+1}=\lim_{q\to \infty }\omega^{q}, \end{array}$$

as the solution of Eq (65).

**Remark 4.** *For the European options and futures, the system* Eq (62) *degenerates to a linear system and can be solved using normal methods*.

What is more, we have the following theorem.

**Theorem 3.** *For* *m* = 1, 2, ⋯, *M*−1, *if* |Δ*t*_*m*_| *is sufficiently small and* $\bar{c}\geq 0$, *then the system matrix of* Eq (65) *is an M-matrix*.

**Proof.** From the definition of *D*(*F*) in Eq (63), it is easy to see that its Jacobian is the following diagonal matrix:

$$\begin{array}{c} J_{D}\left(\omega^{l}\right)=\text{diag}(p^{\prime }\left(F_{1,1}^{m}\right),\cdots ,p^{\prime }\left(F_{N_{S}-1,N_{\delta }-1}^{m}\right)). \end{array}$$

From Eq (64) we know that $p^{\prime }(F_{i,j}^{m})\geq 0$ for all *i* = 1, ⋯, *N*_*S*_−1 and *j* = 1, ⋯, *N*_*δ*_−1. Thus, to show that the system matrix of Eq (65) is an *M*-matrix, it suffices to show that *θE* ^*m*+1^+*G* ^*m*^ is an *M*-matrix.

First, we note that $e_{m,n}^{i,j}\leq 0$ for all *m* ≠ *i*, *n* ≠ *j*, since

$$\begin{array}{c} \frac{b_{i+\frac{1}{2},j}}{S_{i+1}^{\alpha_{i,j}}-S_{i}^{\alpha_{i,j}}}>0,\;\frac{{\bar{b}}_{i,j+\frac{1}{2}}}{e^{{\bar{\alpha }}_{i,j}Y_{j+1}}-e^{{\bar{\alpha }}_{i,j}Y_{j}}}>0 \end{array}$$ (66)

for any *i* and *j*, and for any *α* = *b*/*a* and any $\bar{\alpha }=\bar{b}/\bar{a}$. This is because the function *S* ^*α*^ is increasing when *b* > 0 and decreasing when *b* < 0, and the function $e^{\bar{\alpha }\delta }$ is increasing when $\bar{b}>0$ and decreasing when $\bar{b}<0$. Eq (66) also holds when $b_{i+\frac{1}{2},j}\to 0$, ${\bar{b}}_{i,j+\frac{1}{2}}\to 0$. Furthermore, from Eqs (54)–(56) we know that when ${\bar{c}}_{i,j}\geq 0$, for all *i* = 1, ⋯, *N*_*S*_−1, *j* = 1, ⋯, *N*_*δ*_−1, there holds

$$\begin{array}{ccc} {\left(e_{i,j}^{i,j}\right)}^{m+1} & \geq & |{\left(e_{i-1,j}^{i,j}\right)}^{m+1}\left|+\right|{\left(e_{i,j-1}^{i,j}\right)}^{m+1}\left|+\right|{\left(e_{i,j+1}^{i,j}\right)}^{m+1}\left|+\right|{\left(e_{i+1,j}^{i,j}\right)}^{m+1}|+{\bar{c}}_{i,j}^{m+1}R_{i,j}\\ & = & \sum_{p=1}^{N_{S}-1}\sum_{q=1}^{N_{\delta }-1}\left|{\left(e_{p,q}^{i,j}\right)}^{m+1}\right|+{\bar{c}}_{i,j}^{m+1}R_{i,j}. \end{array}$$

Therefore, *E* ^*m*+1^ is a diagonally dominant with respect to its columns. Hence, from the above analysis, we see that for all admissible *i*, *j*, *E* ^*m*+1^ is a diagonally dominant matrix with positive diagonal elements and non-positive off-diagonal elements. This implies that *E* ^*m*+1^ is an *M*-matrix.

Second, *G* ^*m*^ of the system matrix Eq (65) is a diagonal matrix with positive diagonal entries. In fact, when |Δ*t*_*m*_| is sufficiently small, we have

$$\begin{array}{c} \theta {\bar{c}}_{i,j}R_{i,j}+\frac{R_{i,j}}{-\Delta t_{m}}>0, \end{array}$$

which demonstrates that *θE* ^*m*+1^+*G* ^*m*^ is an *M*-matrix.

Here we emphasize that Theorem 3 implies that the fully discrete system Eq (65) satisfies the discrete maximum principle and the discretization is monotone, such that Eq (65) has a unique solution.

## Numerical results

In this section, some numerical results are presented to demonstrate the efficiency and the usefulness of the numerical method proposed in the above. Furthermore, the varieties of derivatives prices with respect to the spot price, convenience and time, are examined. These are of interest to market participants.

**Test 1**: The European call option with the cash-or-nothing final condition Eq (13).

Parameters: *S*_max_ = 500, *δ*_min_ = −1, *δ*_max_ = −0.25, *T* = 1, *r* = 0.1, *ρ*_*sc*_ = 0.9, *σ*_*s*_ = 0.3, *λ*_*s*_ = −0.2, *σ*_*c*_ = 0.3, *λ*_*c*_ = −0.3, *k*_*s*_ = 0.04, *k*_*c*_ = 0.02, *θ*_*c*_ = −0.2, *ξ* = 0.03, *C*_*s*_ = 21, *K* = 200, *B* = 100.

**Fig 1:**
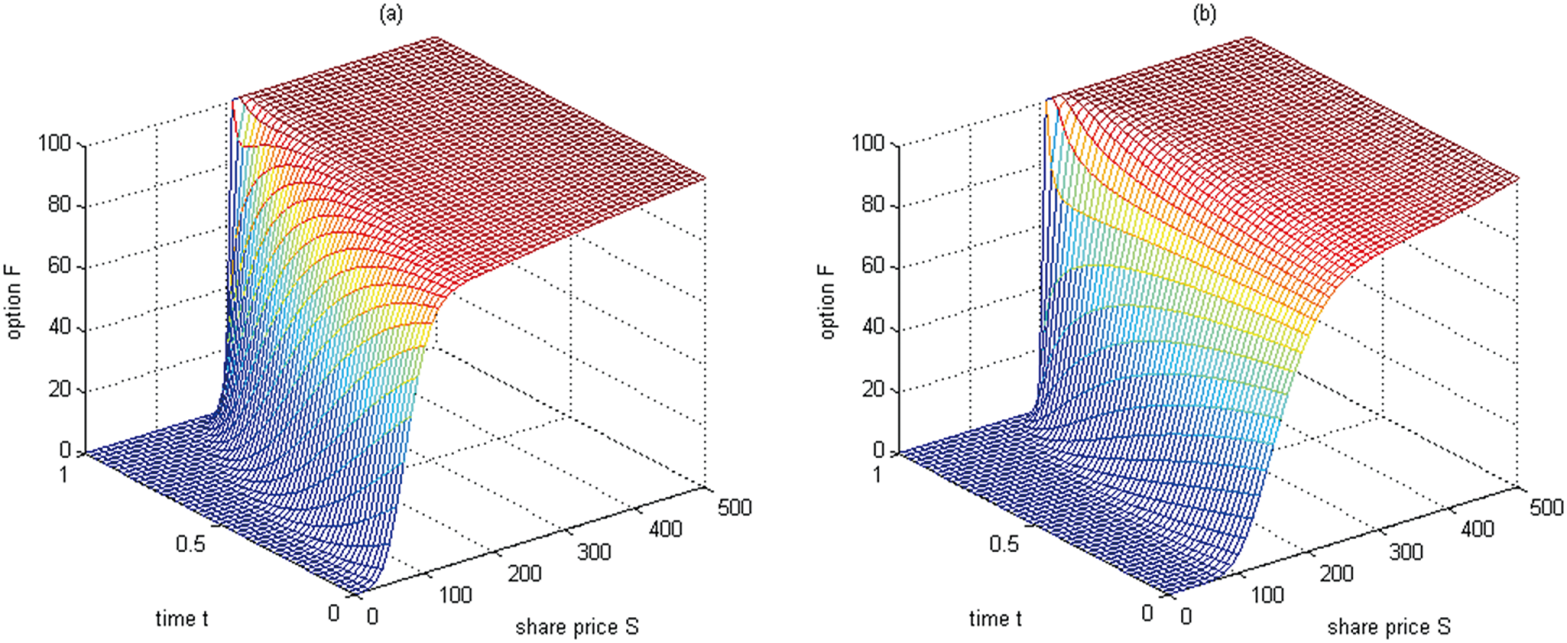
The boundary conditions at *δ* = *δ*_min_ and *δ* = *δ*_max_ for Test 1. (a) the boundary condition *F*(*S*, *δ*_min_, *t*); (b) the boundary condition *F*(*S*, *δ*_max_, *t*)

**Fig 2:**
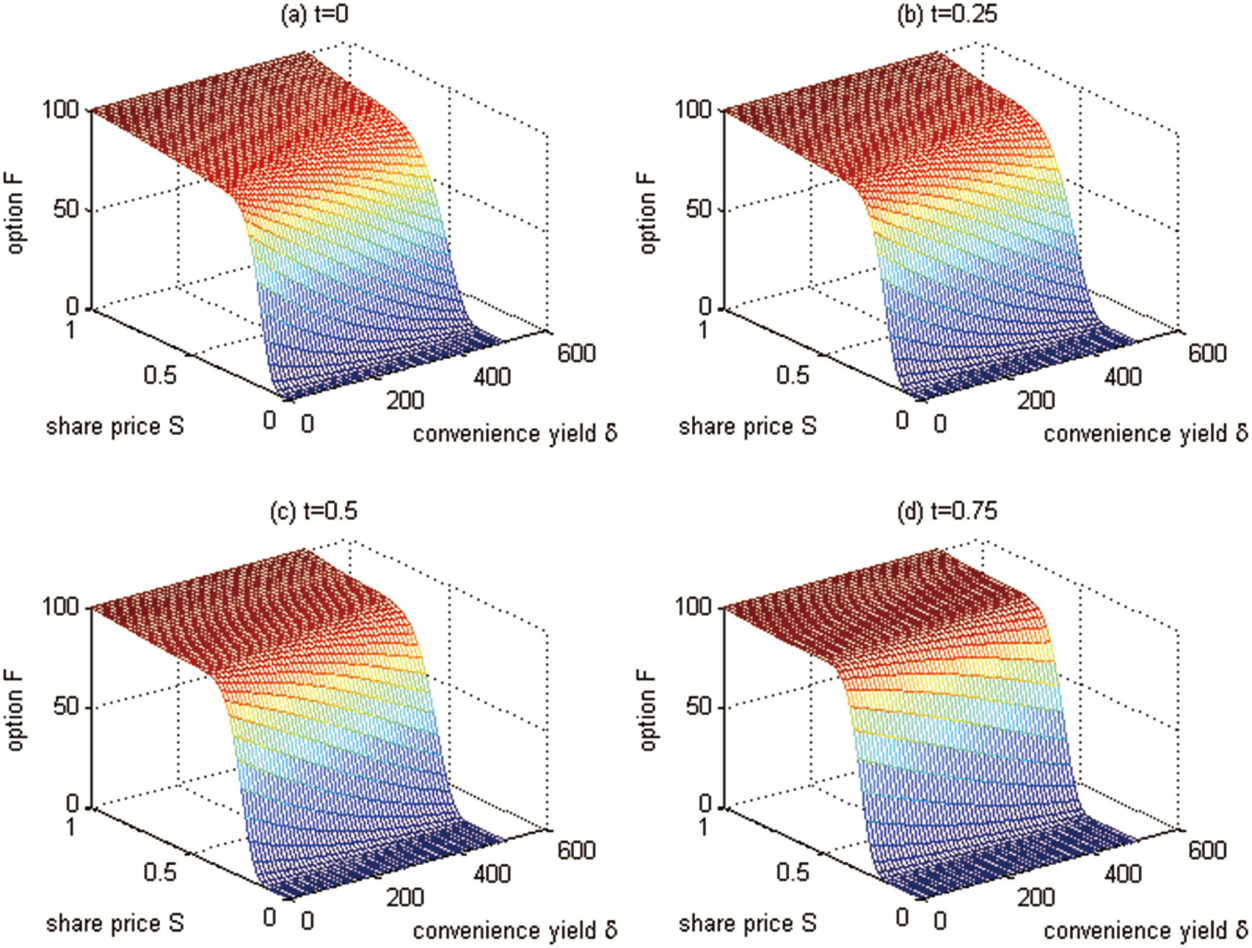
The European cash-or-nothing option values at the time points for Test 1.

To solve the pricing problem numerically, we divide (0, *S*_max_), (*δ*_min_, *δ*_max_), and (0, *T*) uniformly into 50, 50, and 50 sub-intervals, respectively. The boundary conditions are given by Eqs (14), (39), and (40), and the numerical values of these boundary conditions determined by 1D initial-boundary problems are plotted in Fig 1. By means of these initial and boundary conditions, the European option values for Test 1 are computed and the intersection surfaces of different time points are plotted in Fig 2.

We show some option values at some special points in Table 1.

**Table 1:** The European cash-or-nothing option values for some points.

t	*t* = 0	*t* = 0.25	*t* = 0.5	*t* = 0.75
(S, *δ*)				
(240, −1)	93.1038	93.6169	95.0070	96.9608
(240, −0.64)	90.1785	91.4336	93.3164	95.4086
(240, −0.25)	82.5970	83.9493	86.1757	89.9564

Next the convergence rates of the discretization method is gauged. To this end, we define three discrete norms ‖ ⋅ ‖_1,*h*_*S*__, ‖ ⋅ ‖_1,*h*_*δ*__, and ‖ ⋅ ‖_0,*h*_ as follows [42]:

$$\begin{array}{c} {∥v_{h}∥}_{1,h_{S}}^{2}=\sum_{i=1}^{N_{S}-1}\sum_{j=1}^{N_{\delta }-1}S_{i+1/2}^{2}b_{i+1/2,j}h_{\delta_{j}}\frac{S_{i+1}^{\alpha_{i,j}}+S_{i}^{\alpha_{i,j}}}{S_{i+1}^{\alpha_{i,j}}-S_{i}^{\alpha_{i,j}}}{\left(v_{i+1,j}-v_{i,j}\right)}^{2}, \end{array}$$

$$\begin{array}{c} {∥v_{h}∥}_{1,h_{\delta }}^{2}=\sum_{i=1}^{N_{S}-1}\sum_{j=1}^{N_{\delta }-1}\delta_{j+1/2}^{2}{\bar{b}}_{i+1/2,j}h_{S_{i}}\frac{\delta_{j+1}^{{\bar{\alpha }}_{i,j}}+\delta_{j}^{{\bar{\alpha }}_{i,j}}}{\delta_{j+1}^{{\bar{\alpha }}_{i,j}}-\delta_{j}^{{\bar{\alpha }}_{i,j}}}{\left(v_{i,j+1}-v_{i,j}\right)}^{2}, \end{array}$$

$$\begin{array}{c} {∥v_{h}∥}_{0,h}^{2}=\sum_{i=1}^{N_{S}-1}\sum_{j=1}^{N_{\delta }-1}v_{i,j}^{2}R_{i,j}, \end{array}$$

from which we can define the following weighted discrete *H* ^1^-norm:

$$\begin{array}{c} {∥v_{h}∥}_{H^{1}}^{2}={∥v_{h}∥}_{1,h_{S}}^{2}+{∥v_{h}∥}_{1,h_{\delta }}^{2}+{∥v_{h}∥}_{0,h}^{2}. \end{array}$$

In addition, the ratio is defined as:

$$\begin{array}{c} \text{ratio}=\frac{{∥F_{h}^{\Delta t}-F∥}_{\gamma }}{{∥F_{h/2}^{\Delta t/2}-F∥}_{\gamma }}\;\text{with}\;\gamma =\infty \;\text{or}\;H^{1}. \end{array}$$

In Test 1, we employ the numerical solution on the mesh with *N*_*S*_ = 128 = *N*_*δ*_ and *M* = 128 as the “exact” solution *F*, and list the errors in the discrete *L* ^∞^-norm and the weighted discrete *H* ^1^-norm at the final time step *t* = 0 for four consecutive meshes in Table 2. Moreover, the linear regression is used to show that these data obey the basic error estimates as follows:

$$\begin{array}{c} {∥F-F_{h}∥}_{\infty }\approx 0.3136h^{1.0597}\;\text{and}\;{∥F-F_{h}∥}_{H^{1}}\approx 2.0922h^{1.6493}. \end{array}$$

Note that these results are reasonable because of the non-smoothness of the solution due to the pay-off function Eq (13).

**Table 2:** Computed errors in the *L* ^∞^-norm and the *H* ^1^-norm at *t* = 0.

mesh	*L* ^∞^-norm	ratio	*H* ^1^-norm	ratio
4×4×4	67.1832		6.2069e+003	
8×8×8	17.1719	3.9124	1.6034e+003	3.8711
16×16×16	10.8381	1.5844	712.2445	2.2512
32×32×32	8.1675	1.3270	204.2685	3.4868
64×64×64	2.4752	3.2997	57.2606	3.5673

From Figs 1, 2, and Table 1 we can conclude that

1. The European cash-or-nothing option values are higher when the option comes to maturity. This is acceptable in that the European option can be only exercised at maturity, and one can not get more benefits when holding a European option that is not closed to maturity, which is different from the American option.
2. The greater convenience yield *δ*, the lower the option value. This can be explained as follows: since the convenience yield *δ* measures the benefits from holding the carbon emission permits rather than the derivatives contract, people will hold more carbon emissions spots when facing a higher convenience yield, which leads to the demand reduction for derivatives and the option price down.
3. As the value of carbon emission spots rises, the value of call option also rises. This is because the holder of call option will get more chances for benefits from the derivatives. This result is similar to the general call options.

**Test 2**: The American call option with the ramp payoff final condition Eq (12).

Parameters: *S*_max_ = 500, *δ*_min_ = −1, *δ*_max_ = −0.25, *T* = 1, *r* = 0.1, *ρ*_*sc*_ = 0.9, *σ*_*s*_ = 0.3, *λ*_*s*_ = −0.2, *σ*_*c*_ = 0.3, *λ*_*c*_ = −0.3, *k*_*s*_ = 0.04, *k*_*c*_ = 0.02, *θ*_*c*_ = −0.2, *ξ* = 0.03, *C*_*s*_ = 21, *K* = 200, *λ* = 100, *k* = 16.

**Fig 3:**
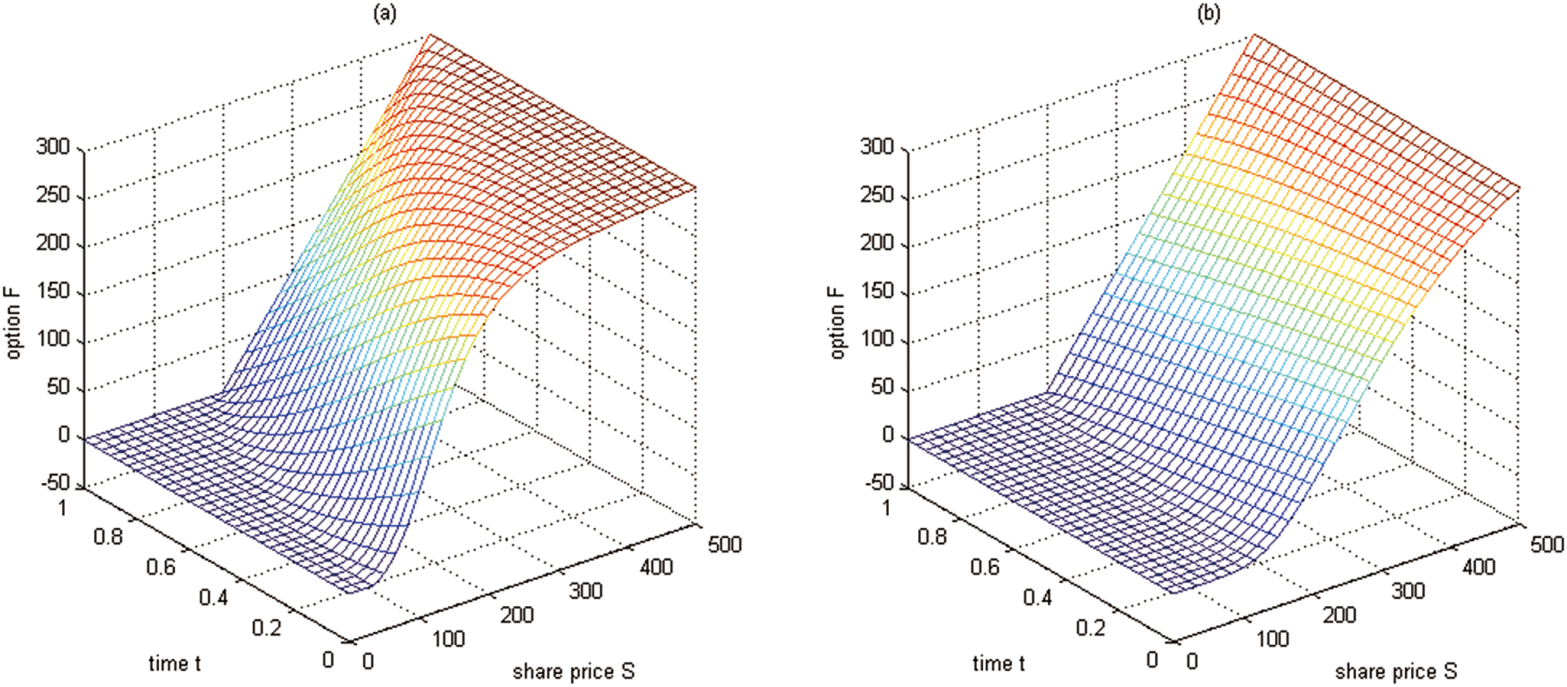
The boundary conditions at *δ* = *δ*_min_ and *δ* = *δ*_max_ for Test 2. (a) the boundary condition *F*(*S*, *δ*_min_, *t*); (b) the boundary condition *F*(*S*, *δ*_max_, *t*)

**Fig 4:**
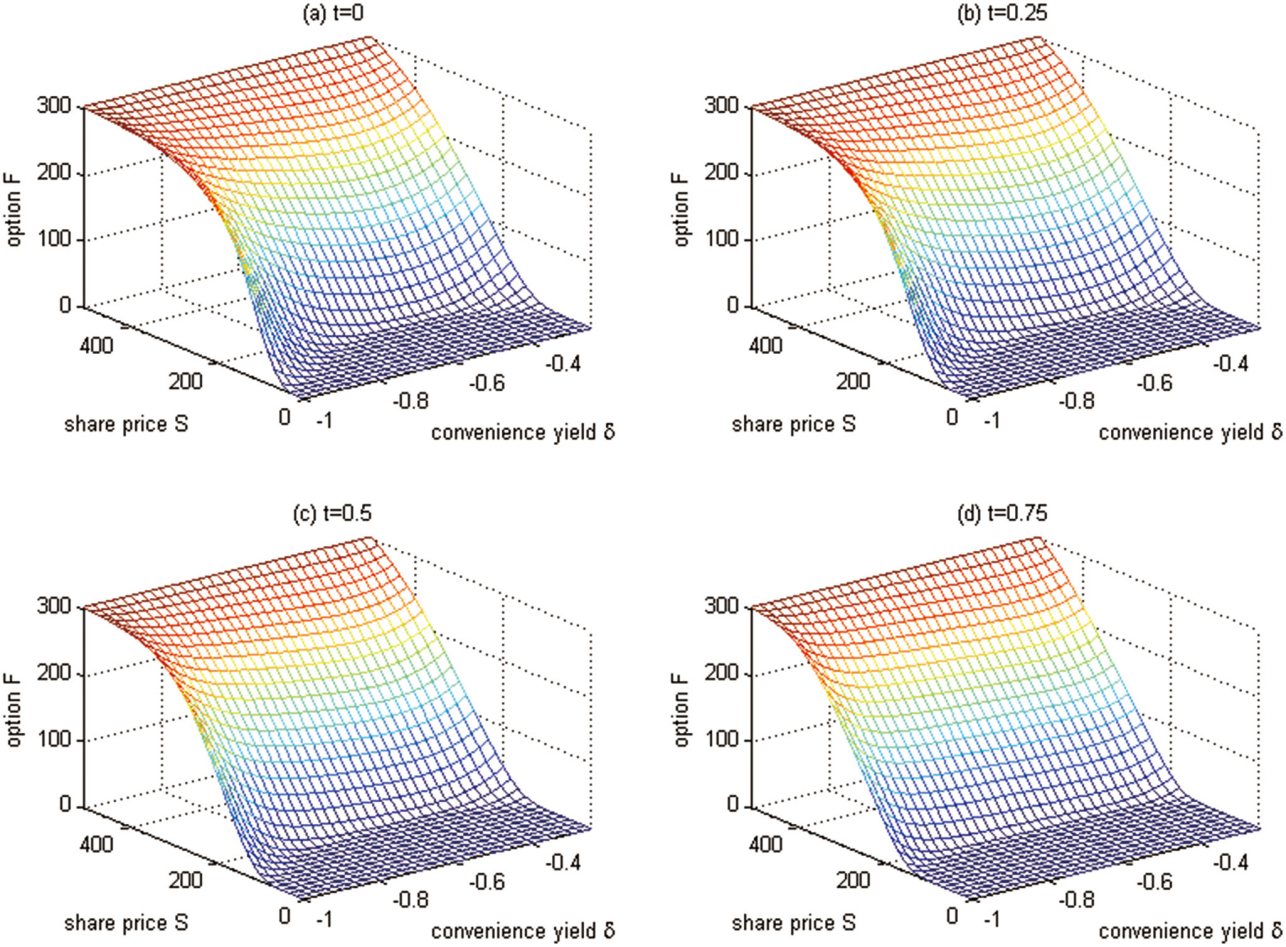
American call option values at time points for Test 2.

To solve the American call option with the above parameters in Test 2, we divide (0, *S*_max_), (*δ*_min_, *δ*_max_), and (0, *T*) uniformly into 30, 30, and 30 sub-intervals, respectively. The final and boundary conditions are given by Eqs (20), (21), (39), and (40), and the numerical values of these boundary conditions determined by the 1D initial-value problems are plotted in Fig 3. According to these initial and boundary conditions, the American call option problem for Test 2 is solved and the intersection surfaces of different time points are depicted in Fig 4. We also show some option values at some special points in Table 3.

**Table 3:** American call option values for some points.

t	*t* = 0	*t* = 0.25	*t* = 0.5	*t* = 0.75
(S, *δ*)				
(100, −0.6)	0	0	0	0
(200, −0.6)	48.3564	36.5279	21.7587	8.9085
(300, −0.6)	158.9589	152.3870	139.0894	115.0307
(200, −1)	240.3643	206.7208	137.2206	54.2146
(200, −0.6)	48.3564	36.5279	21.7587	10.5838
(200, −0.3)	50.8563	39.9947	26.0251	11.6181

From Figs 3, 4 and Table 3, we have the conclusions as follows:

1. The American vanilla call option values are lower when the option comes to maturity. An American option can be exercised at any time between the date of purchase and the expiration date. As the option comes to maturity, the benefits from the early exercise of option are reduced, and then the option prices decrease. This is the same as the popular financial option contracts.
2. Unlike European options, for American options the greater convenience yield *δ* does not mean a lower option value (see the last two lines of Table 3). As we all have known, the value of American option contains an early exercise premium. Therefore, although the greater convenience yield reduces the demand for derivatives and decreases the option price, the early exercise premium may also increase the option price oppositely. So, there is no certain relationship between the convenience yield and the option value.
3. As the value of carbon emission spots rises, the value of the call option also goes up. This is because the holder of the call option can get more chances to benefit from it. This result is similar to general call options.

**Fig 5:**
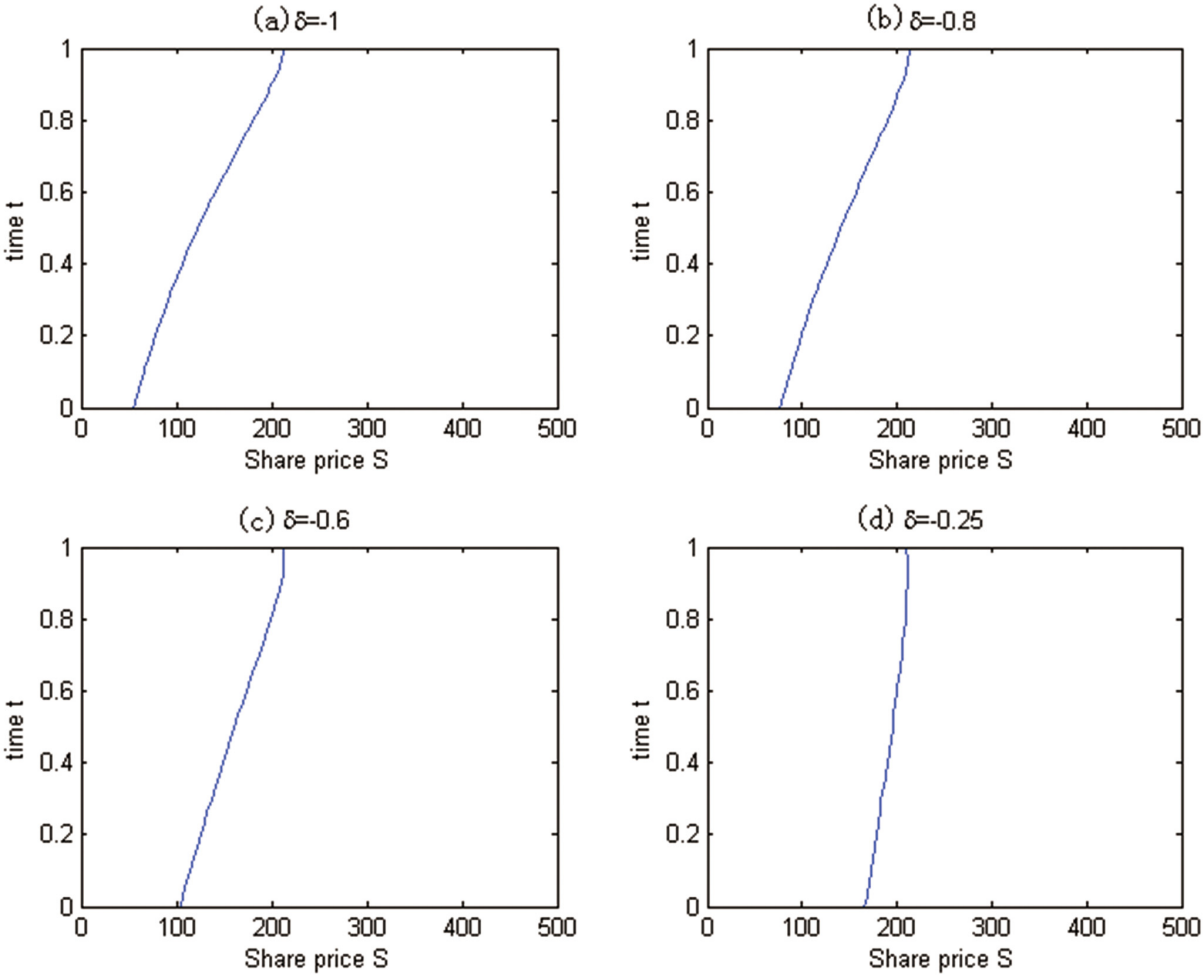
Optimal exercise boundaries about different *δ* for Test 2.

Financial engineers could pay more attention to the optimal exercise boundary compared with the option value in the case of American option. We determine the boundary *S* ^⋆^(*t*) by Eq (16), and the results are plotted in Fig 5. The blue line is the optimal exercise boundary, the left of the blue line is the continuation region, and the right of the blue line is the stopping region. We can clearly see that as the convenience yield increases, the stopping region becomes smaller. Note that our pricing model is different from the classical Black-Scholes model in details, and this result should be reasonable.

**Test 3**: The futures with the final condition Eq (17).

Parameters: *S*_max_ = 500, *δ*_min_ = −1, *δ*_max_ = −0.25, *T* = 1, *r* = 0.1, *ρ*_*sc*_ = 0.9, *σ*_*s*_ = 0.3, *λ*_*s*_ = −0.2, *σ*_*c*_ = 0.3, *λ*_*c*_ = −0.3, *k*_*s*_ = 0.04, *k*_*c*_ = 0.02, *θ*_*c*_ = −0.2, *ξ* = 0.03, *C*_*s*_ = 21.

**Fig 6:**
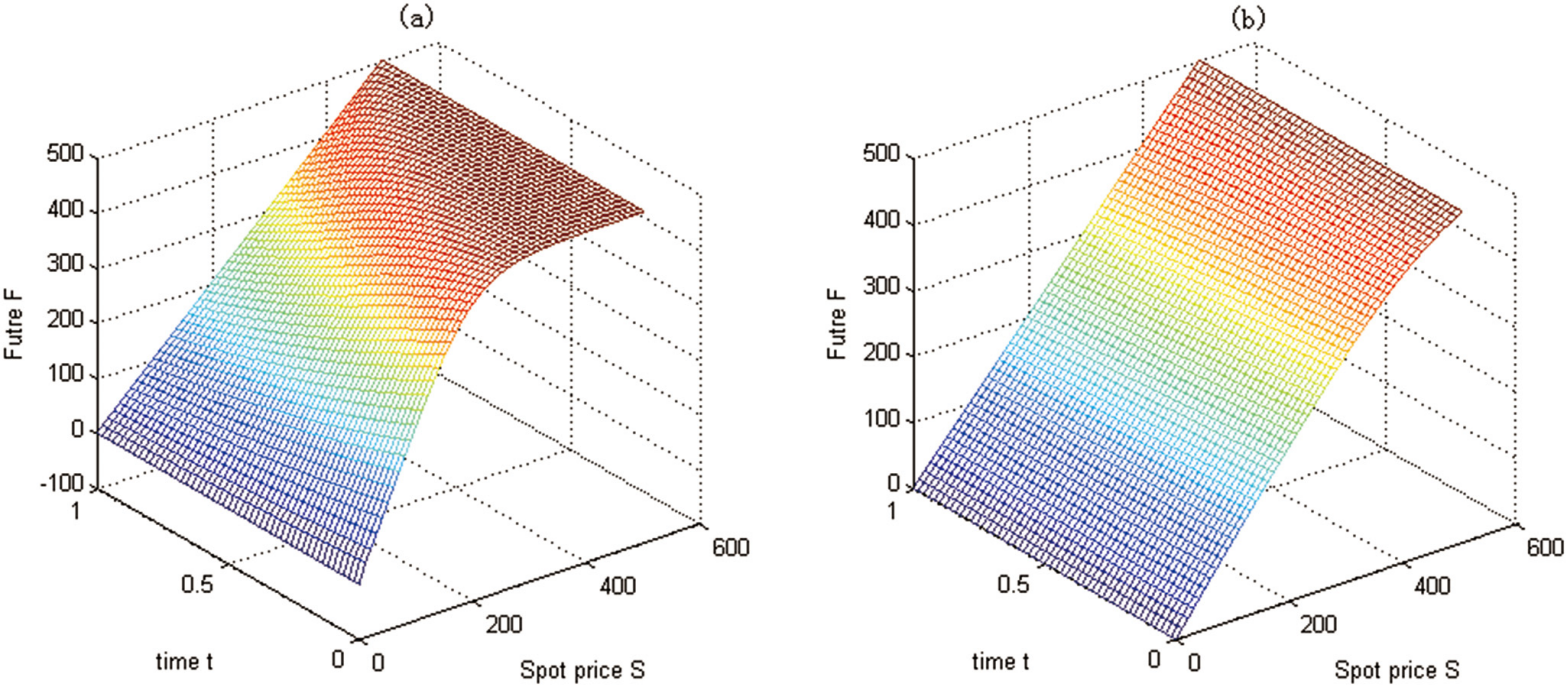
The boundary conditions at *δ* = *δ*_min_ and *δ* = *δ*_max_ for Test 3. (a) the boundary condition *F*(*S*, *δ*_min_, *t*); (b) the boundary condition *F*(*S*, *δ*_max_, *t*)

**Fig 7:**
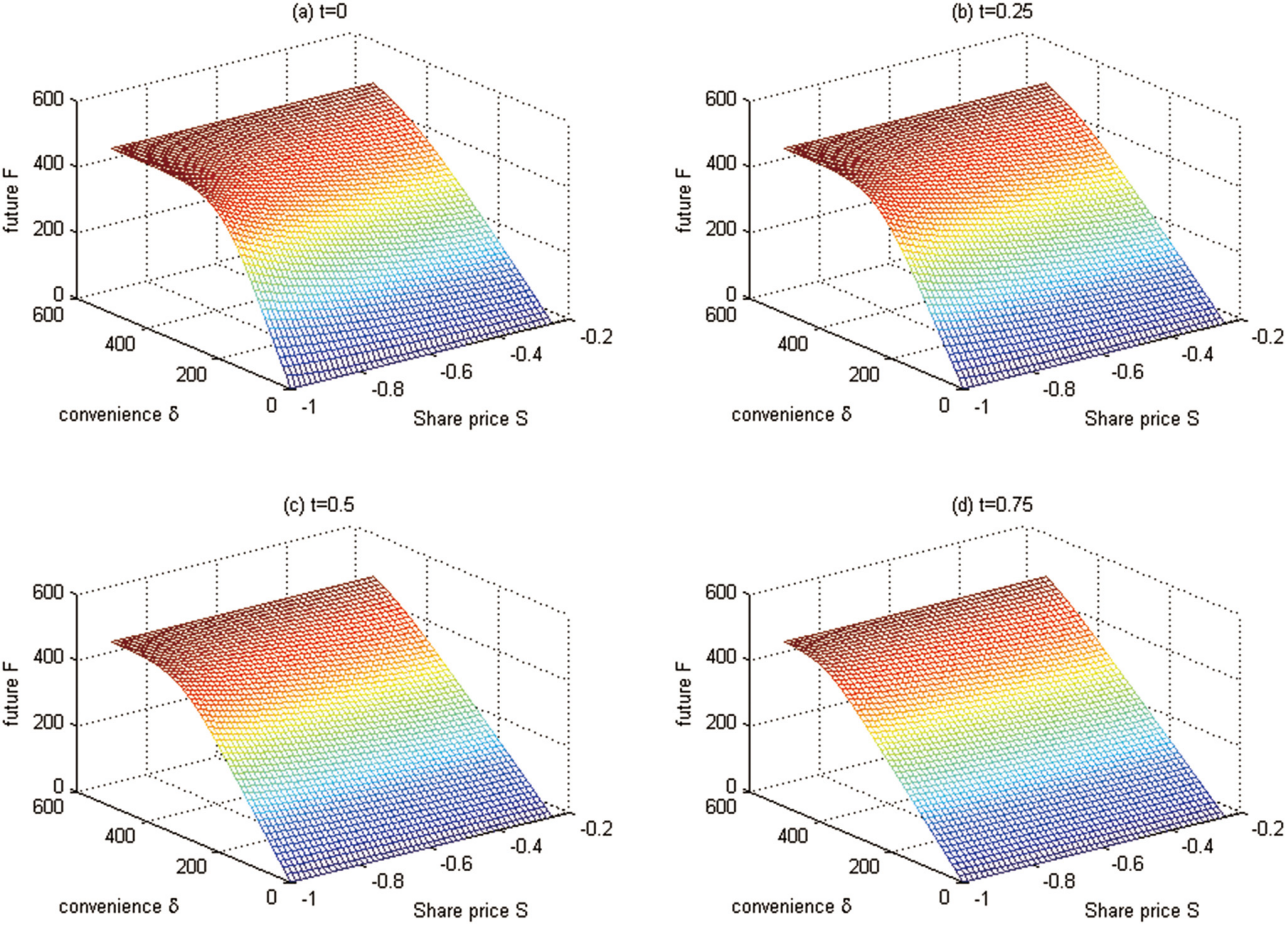
Futures values at time points for Test 3.

To solve the futures with the above parameters in Test 3, we divide (0, *S*_max_), (*δ*_min_, *δ*_max_), and (0, *T*) uniformly into 50, 50, and 50 sub-intervals, respectively. The boundary conditions are given by Eqs (14), (39), and (40), and the numerical values of these boundary conditions determined by the 1D initial-value problems are plotted in Fig 6. According to these initial and boundary conditions, the futures problem for Test 3 is solved, and the intersection surfaces of different time points are depicted in Fig 7.

Some futures values at some special points are shown in Table 4.

**Table 4:** Futures values for some points.

t	*t* = 0	*t* = 0.25	*t* = 0.5	*t* = 0.75
(S, *δ*)				
(250, −1)	477.8765	451.4826	393.4370	322.6292
(250, −0.82)	410.0061	372.1902	327.5114	282.7984
(250, −0.61)	354.9645	322.1501	293.8633	267.3085

From Figs 6, 7 and Table 4, we have the conclusions as follows:

1. The futures prices are lower when the futures contract comes to the maturity. This is because the futures prices converge to the spot prices when the futures contract comes to the maturity. With time approaching to the maturity date, the uncertainty, which should increase the futures prices, is reducing.
2. The greater the convenience yield *δ*, the lower the futures value. This can be explained as follows: since the convenience yield *δ* measures the benefits from holding the carbon emission permits rather than the derivatives contract, people will hold more carbon emission spots when facing a higher convenience yield, which leads to the demand reduction for derivatives and the futures price down.
3. As the value of carbon emission spots goes up, the value of futures also rises. This is because the holder of futures will get more chances to benefit from the derivatives. This result is similar to general futures.

## Discussions

In this section, we examine the sensitivity of above results to the parameters variations. There are four parameters which are likely to have an impact on the price of emission allowance derivatives.

**Fig 8:**
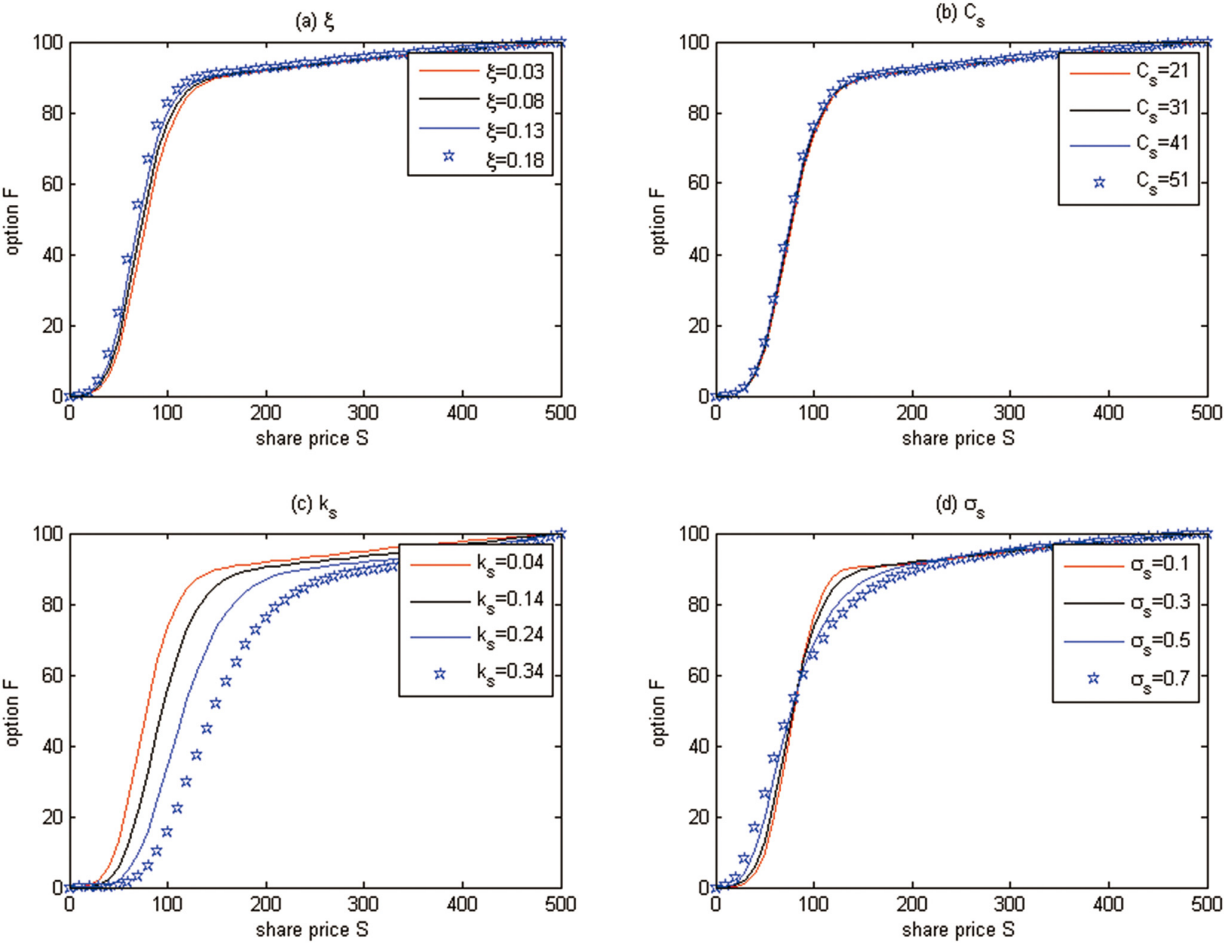
The effects of some parameters on the European option prices for Test 1.

**Discussion 1.** We examine the effects of some parameters on the European cash-or-nothing option prices in Fig 8, where we fix *t* = 0 and *δ* = −1.

From Fig 8 we can see that

- The option price increases with the higher growing rate of marginal abatement costs. The higher growing rate of marginal abatement costs will make the abatement costs change quickly and the enterprises will live in a more uncertain world when they must choose investing on clean technology or trading the emission permits. To eliminate this uncertainty, enterprises will buy more derivatives such as options, which leads to the option value increasing.
- The effect of the marginal abatement costs is negligible. The purpose to trade the emission permits is just to hedge the risk of marginal abatement costs, and if marginal abatement costs increase, people will hold more emission permit spots, and the spots price will increase and vice versa. So, it is natural that the option value does not change following the movement of marginal abatement costs.
- Increasing the speed of mean-reversion *k*_*s*_ reduces the option prices. This is because emission permit prices have a stronger tendency towards the mean value, the price risk of emission permits decreases, and then people need not to hold many derivatives to hedge the risk. The decrease of demand will reduce the option prices.
- The effect of the volatility *σ*_*s*_ is irregular compared with the classical Black-Scholes model. In the Black-Scholes world, the higher volatility of underlying assets increases the risk of investment, and the derivatives price will increase. In our model, since the stochastic convenience yield is considered, the effect of volatility on option prices may be counteracted by the stochastic convenience yield. So, there is no certain relationship between the volatility and the option price when there is a stochastic convenience yield.

Note that the above sensitivity analysis results are also satisfied by the following American option and futures, as they are all the derivatives used to hedge the price risk.

**Fig 9:**
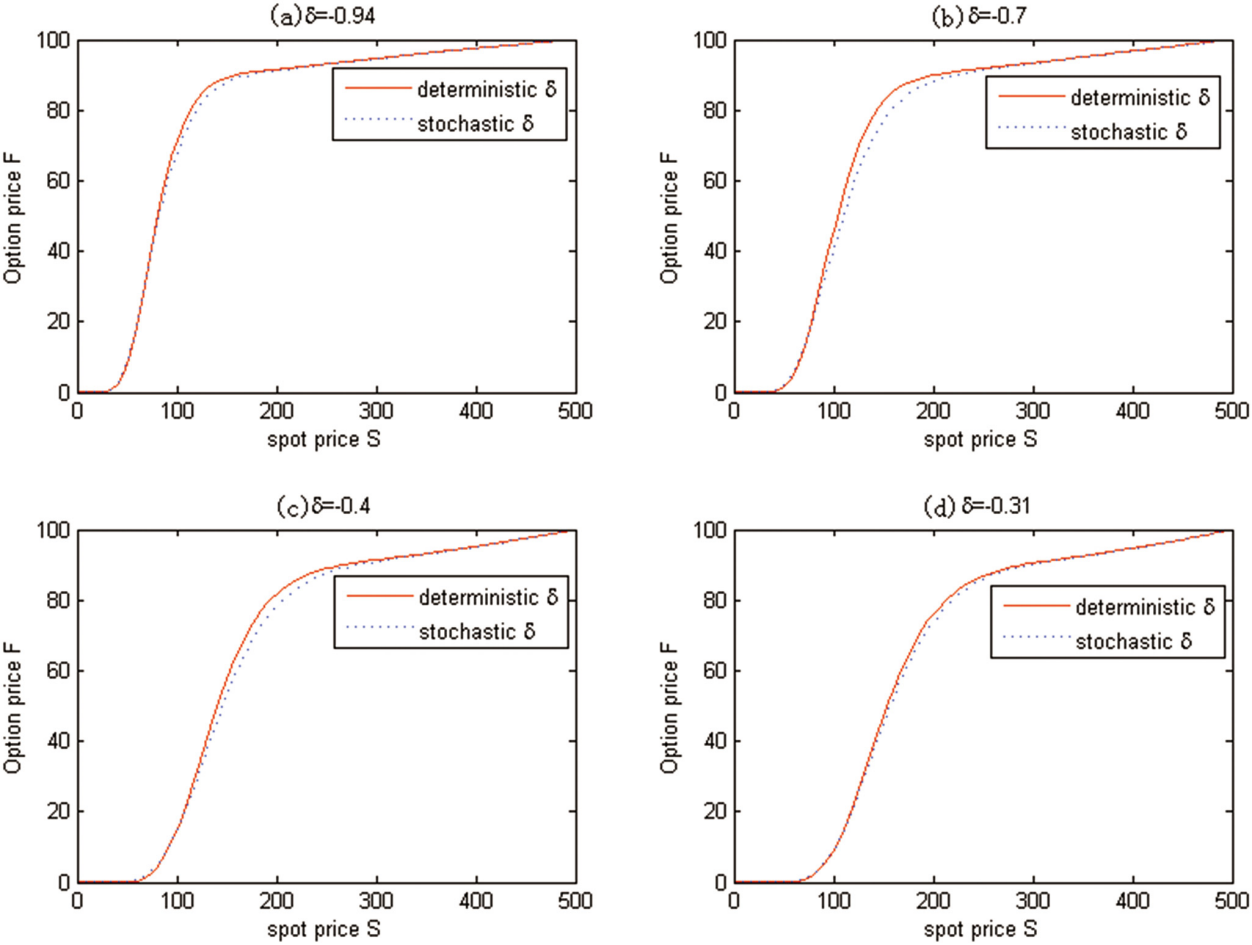
The effects of stochasticity of convenience yield on the European option prices for Test 1.

Additionally, the effects of stochasticity of convenience yield on the valuations of carbon European options are also examined in Fig 9, where *t* = 0 is fixed. Note that the solid line states the deterministic convenience yield simulation results and the dash line represents the stochastic ones.

**Fig 10:**
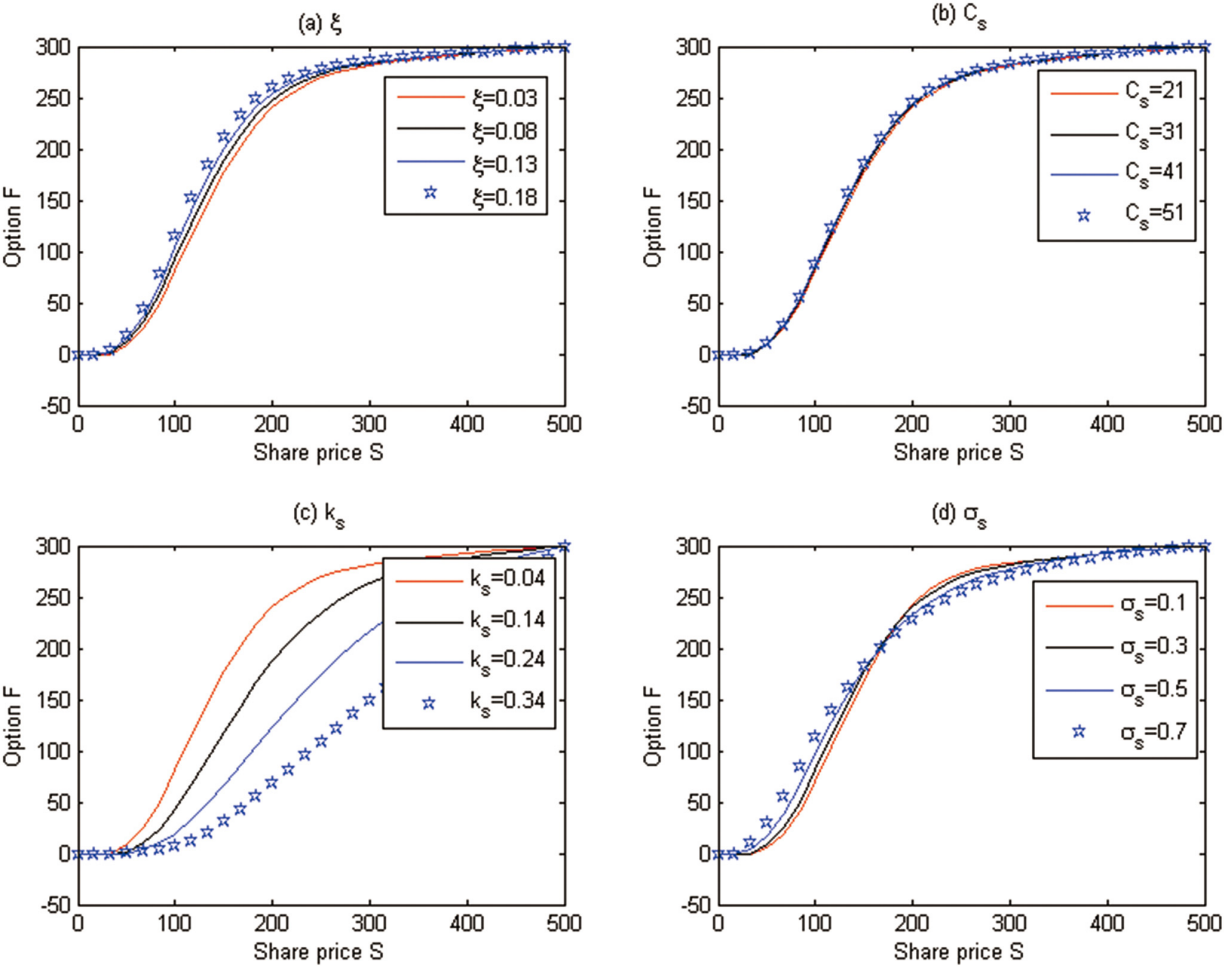
The effects of some parameters on the American option prices for Test 2.

**Discussion 2.** The effects of some parameters on the American option price are examined in Fig 10, in which *t* = 0 and *δ* = −1.

From Fig 10, again we can see that

- The option price increases with the higher growing rate of marginal abatement costs.
- The effect of the marginal abatement costs is negligible.
- Increasing the speed of mean-reversion *k*_*s*_ reduces the option prices.
- The effect of the volatility *σ*_*s*_ is irregular compared with the classical Black-Scholes model.

**Fig 11:**
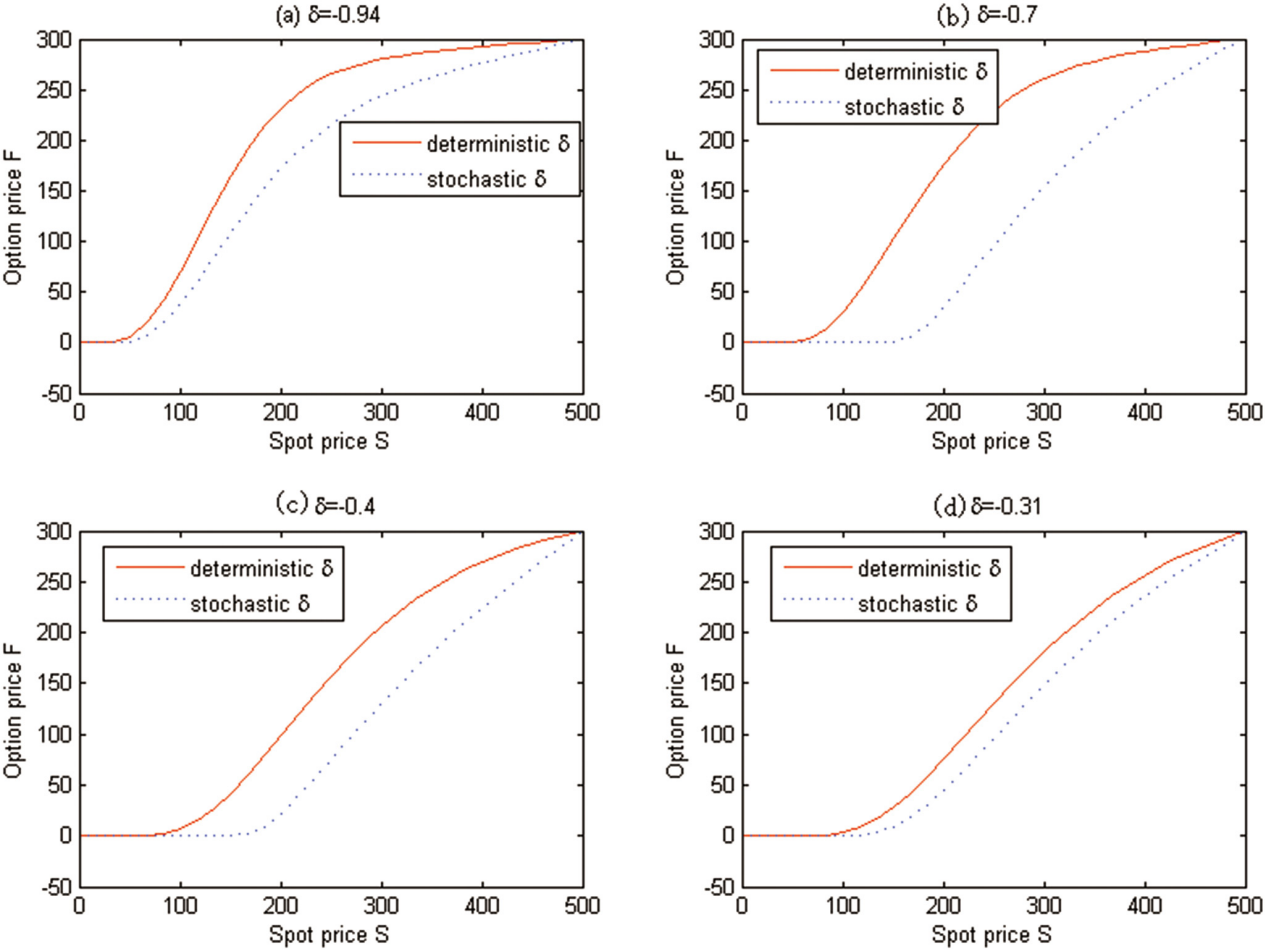
The effects of stochasticity of convenience yield on the American option prices for Test 2.

Similarly, the effects of stochasticity of convenience yield on the valuation of carbon American option are also examined in Fig 11, where *t* = 0 is fixed. Note that the solid line states the deterministic convenience yield simulation results and the dash line represents the stochastic ones.

**Fig 12:**
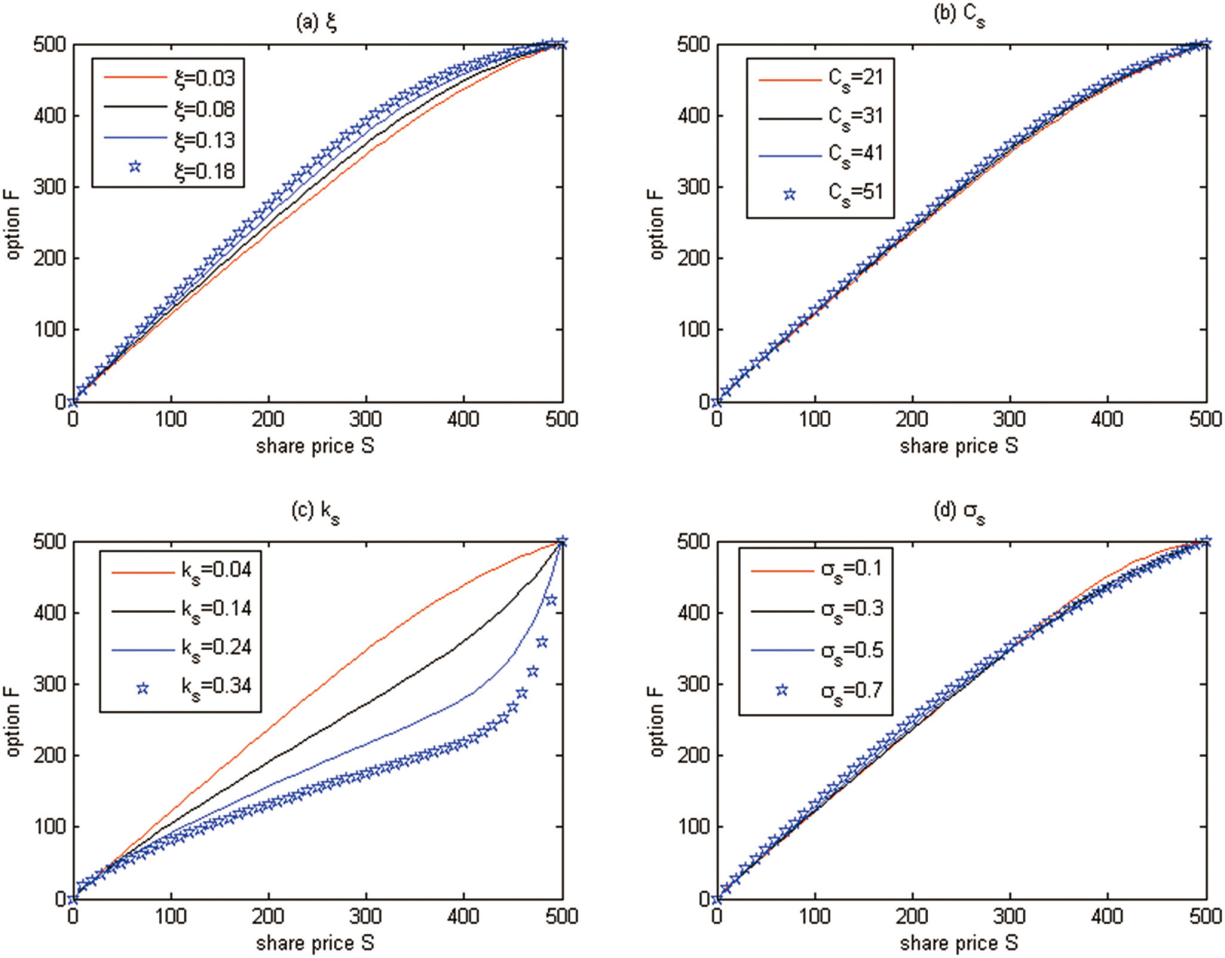
The effects of some parameters on the futures price for Test 3.

**Discussion 3.** The effects of some parameters on the futures prices are examined in Fig 12, in which *t* = 0 and *δ* = −1.

From Fig 12 we can see that

- The futures price increases with the higher growing rate of marginal abatement costs, which increases the uncertainty.
- The effect of the marginal abatement costs is negligible.
- Increasing the speed of mean-reversion *k*_*s*_ reduces the futures price, because the emission permits price trends to the mean value more quickly, and the uncertainty of emission permits price would become smaller.
- There is no certain effect of the volatility on futures prices.

**Fig 13:**
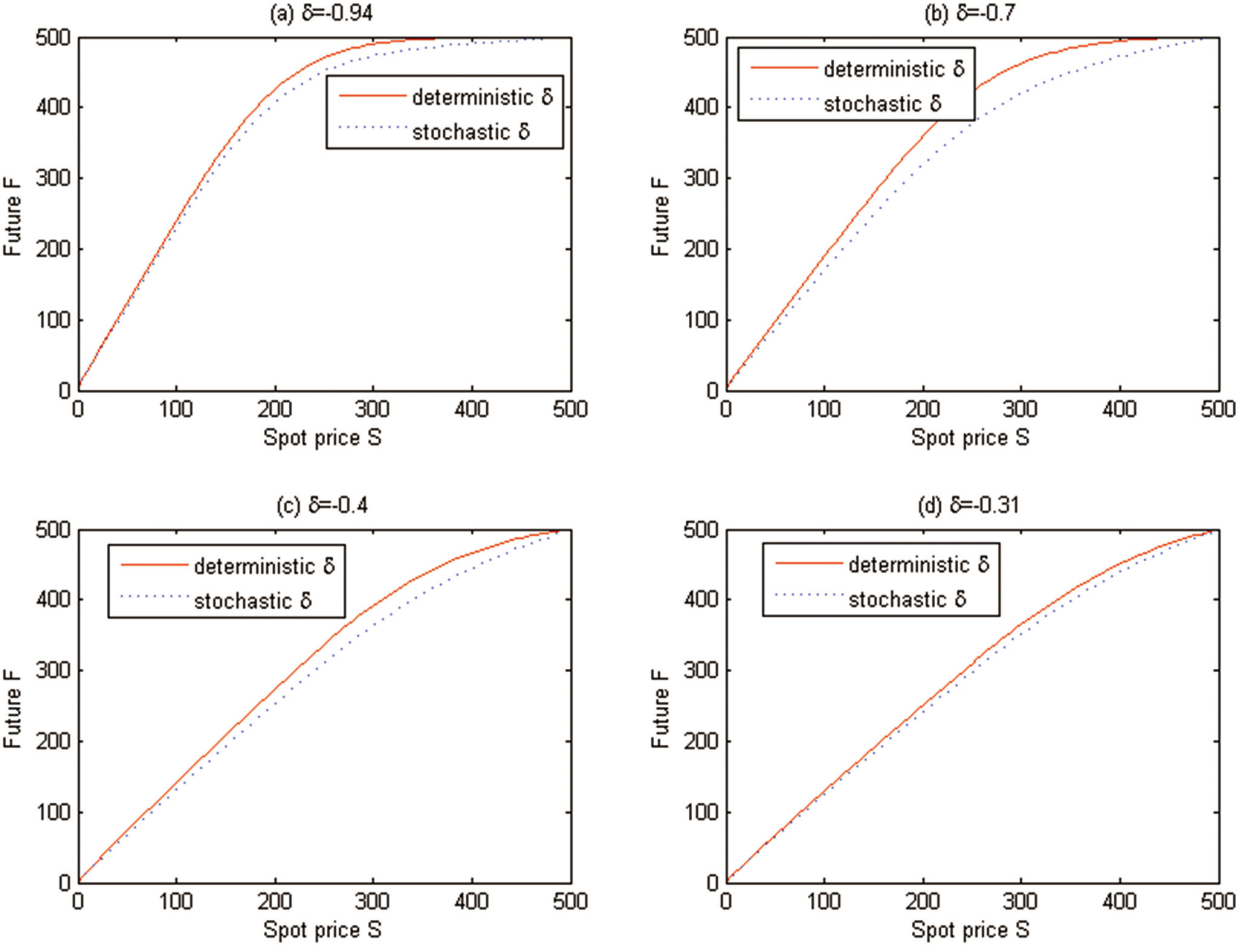
The effects of stochasticity of convenience yield on the futures prices for Test 3.

Once again, the effects of stochasticity of convenience yield on the valuations of carbon futures are examined in Fig 13 with the fixed *t* = 0. Note that the solid line corresponds to the deterministic convenience yield simulation results and the dash line represents the stochastic ones.

From the above discussions, we can summarize some experiences about risk management. Firstly, as the Kyoto Protocol came into force in 2005, several countries are expanding their investment scales on the new technologies to reduce carbon emissions, and thus the emission allowance derivatives are also becoming a risk hedging tool for market participants. Secondly, a firm may not be scarce to the emission allowance derivatives when it can adjust its marginal abatement costs well. Finally, the impact of volatility of carbon emission allowances price is difficult to measure when a stochastic convenience yield is involved.

## Concluding remarks

This paper presents a methodology for modelling and computing the valuation of carbon derivatives with stochastic convenience yields. The principle of absence of arbitrage opportunities and the stochastic calculus are used to develop the mathematical model, the partial differential equation, satisfied by carbon derivatives. For American options, we formulate the pricing problem to a linear parabolic variational inequality, and develop a power penalty method to solve it. Then, a so-called fitted finite volume method is designed to solve the nonlinear partial differential equation resulting from the power penalty method, which governs the futures, European and American option valuation.

Also, we discuss the effects of the stochastic convenience yield on the prices of carbon derivatives in details. First of all, a greater convenience yield means a lower European option price and futures price while it does not mean a lower American option price. Therefore, the property of optimal exercise boundary makes the American option quite different from the European option. Furthermore, from the sensitivity analysis we can see that (*a*) the derivatives price increases with the higher growing rate of marginal abatement costs; (*b*) the effect of the marginal abatement costs is negligible; (*c*) increasing the speed of mean-reversion *k*_*s*_ reduces the derivatives price; (*d*) there is no certain effect of the volatility on derivatives price compared with the classical Black-Scholes model.

The carbon market participants, such as investors, hedgers and arbitragers, may get help from our theoretical results, and they can work out better hedging strategies, adjust portfolio structures and strengthen their capabilities to manage risks. We expect that our work can emphasize the importance of the convenience yield concept for the emission allowance pricing. At the same time, we also anticipate that our derivatives pricing methodology from the perspective of partial differential equations combined with numerical methods can make a few contributions to the development of carbon market.
